# *Pianp* deficiency links GABA_B_ receptor signaling and hippocampal and cerebellar neuronal cell composition to autism-like behavior

**DOI:** 10.1038/s41380-019-0519-9

**Published:** 2019-09-11

**Authors:** Manuel Winkler, Siladitta Biswas, Stefan M. Berger, Moritz Küchler, Laurens Preisendörfer, Myeongjeong Choo, Simon Früh, Pascal D. Rem, Thomas Enkel, Bernd Arnold, Dorde Komljenovic, Carsten Sticht, Sergij Goerdt, Bernhard Bettler, Oliver von Bohlen und Halbach, Dusan Bartsch, Cyrill Géraud

**Affiliations:** 1 Department of Dermatology, Venereology, and Allergology, University Medical Center and Medical Faculty Mannheim, Heidelberg University, and Center of Excellence in Dermatology, Mannheim, Germany; 2 Department of Molecular Biology, Central Institute of Mental Health and Medical Faculty Mannheim, Heidelberg University, Mannheim, Germany; 3 Institute of Anatomy and Cell Biology, University Medicine Greifswald, Greifswald, Germany; 4 Department of Biomedicine, Institute of Physiology, University of Basel, Basel, Switzerland; 5 Division of Molecular Immunology, German Cancer Research Center (DKFZ), Heidelberg, Germany; 6 Division of Medical Physics in Radiology, German Cancer Research Center (DKFZ), Heidelberg, Germany; 7 Center for Medical Research, Medical Faculty Mannheim, Heidelberg University, Mannheim, Germany; 8 European Center for Angioscience, Medical Faculty Mannheim, Heidelberg University, Mannheim, Germany; 9 Section of Clinical and Molecular Dermatology, Medical Faculty Mannheim, Heidelberg University, Mannheim, Germany

**Keywords:** Neuroscience, Molecular biology, Genetics

## Abstract

Pianp (also known as Leda-1) is a type I transmembrane protein with preferential expression in the mammalian CNS. Its processing is characterized by proteolytic cleavage by a range of proteases including Adam10, Adam17, MMPs, and the γ-secretase complex. Pianp can interact with Pilrα and the GB1a subunit of the GABA_B_ receptor (GBR) complex. A recent case description of a boy with global developmental delay and homozygous nonsense variant in *PIANP* supports the hypothesis that PIANP is involved in the control of behavioral traits in mammals. To investigate the physiological functions of Pianp, constitutive, global knockout mice were generated and comprehensively analyzed. Broad assessment did not indicate malformation or malfunction of internal organs. In the brain, however, decreased sizes and altered cellular compositions of the dentate gyrus as well as the cerebellum, including a lower number of cerebellar Purkinje cells, were identified. Functionally, loss of *Pianp* led to impaired presynaptic GBR-mediated inhibition of glutamate release and altered gene expression in the cortex, hippocampus, amygdala, and hypothalamus including downregulation of *Erdr1*, a gene linked to autism-like behavior. Behavioral phenotyping revealed that *Pianp* deficiency leads to context-dependent enhanced anxiety and spatial learning deficits, an altered stress response, severely impaired social interaction, and enhanced repetitive behavior, which all represent characteristic features of an autism spectrum disorder-like phenotype. Altogether, *Pianp* represents a novel candidate gene involved in autism-like behavior, cerebellar and hippocampal pathology, and GBR signaling.

## Introduction

Ajap1 and Pianp (initially described as Leda-1) constitute a family of type I transmembrane proteins preferentially expressed in the mammalian central nervous system (CNS) [1, 2]. *Ajap1*, contained in chromosomal region 1p36 [3], was found to be frequently deleted in neuroblastoma and oligodendroglioma and thus represents a tumor suppressor gene in these tumors [4]. Notably, Isidor et al. [5] described a child with a complex constitutional subtelomeric 1p36.3 deletion/duplication that has intellectual disability (ID) and neonatal neuroblastoma indicating that *Ajap1* may have important functions in the brain beyond tumor suppression. In addition, *Ajap1* was identified as a new susceptibility locus for migraine [6] and as a novel candidate gene for the treatment response to risperidone in schizophrenia [7].

In contrast to Ajap1, Pianp is less well studied. Although most strongly expressed in the brain, Pianp was initially identified in rat liver sinusoidal endothelial cells [8]. Similar to Ajap1, Pianp also sorts to the basolateral domain of the plasma membrane and alters E-cadherin processing in polarized epithelial cells [8, 9]. Pianp itself also undergoes posttranslational proteolytic processing by Furin-like pro-protein convertases, Matrix-metalloproteinases (MMPs) as well as Adam10 or Adam17, and the γ-secretase complex [10, 11]. In addition, Pianp was shown to be expressed in lymphoid organs and bone marrow-derived macrophages of BALB/c mice [2]. In line with this, Pianp was identified as a ligand of immune inhibitory receptor Pilrα in vitro and it has been shown that glycosylation of Pianp is necessary for this interaction [1]. Pianp and Pilrα are counter regulated upon LPS stimulation of murine macrophages and MMPs are responsible for LPS-mediated downregulation of Pianp in these cells [2].

With regard to its putative functions in the brain, Anazi et al. [12] recently described a boy with a homozygous nonsense variant in *PIANP* upon performing an analysis of the morbid genome of human ID. This boy was 1 year and 8 months old, had dysmorphic facial and acral features, central hypotonia, and showed a delay of global development, as he was unable to walk and did not use simple words such as mama.

Autism spectrum disorders (ASD) represent a complex group of neurodevelopmental disorders that are characterized by impaired social behavior and communication as well as repetitive behavior and restricted interests. Although ID is not a prerequisite of ASD, both tend to be associated and correlate in their severity [13]. Interestingly, the cerebellum appears to be a key region affected by autism and Purkinje cells, GABAergic neurons located in the cerebellar cortex, have been shown to be reduced in number and density in ASD [14]. The role of Purkinje cells has also been underscored by the description of autism-like neuroanatomic alterations and behaviors in mice with Purkinje cell-specific deficiency of either *Tsc1* [15], *Tsc2* [16], or *Shank2* [17]. Although there is no uniform classification for endophenotypes of ASD, it was recently proposed that an ASD-associated gene cluster expressed in Purkinje cells correlated with ID-free ASD in comparison to an ASD-associated gene cluster expressed in the neocortex, which was related to ID-associated ASD [18].

Recently, it was shown that Pianp, Ajap1, and App form three distinct GABA_B_ receptor (GBR) complexes by binding to the N-terminal sushi-domain of GB1a, a subunit of presynaptic GBRs [19, 20]. While App is necessary for axonal GBR expression, Ajap1 and Pianp are not required for axonal transport [20]. Lack of App results in a significant deficit in presynaptic inhibition of neurotransmitter release by the GBR agonist baclofen [20]. The role of Pianp in presynaptic GBR functioning has not been thoroughly analyzed yet. GBRs are key regulators of synaptic transmission in the brain [21]. Presynaptic GBRs inhibit the release of a variety of neurotransmitters, including glutamate and GABA [21]. GABAergic signaling is altered in a variety of diseases including neurodevelopmental disorders such as ASD [22, 23]. Despite the fact that GBRs are not yet described to be directly involved in ASD, they are shown to influence other neurodevelopmental disorders such as fragile X syndrome [24]. However, as Purkinje cells have emerged as key cells mediating ASD-like phenotypes in mice [14–17], and Purkinje cells represent the neuronal cell type with the highest levels of GBRs [25], involvement of GBR signaling in autism-like behavior appears reasonable. Although deficiency of the main subunits of GBRs in mice did not reveal autism-like behaviors [26], there is evidence that GABAergic signaling is functionally impaired in ASD despite normal GABA receptor availability [22, 23, 27]. Such impairment may be mediated by modifications of GABA receptor interactions with associated proteins.

In order to comprehensively analyze the physiological functions of Pianp in vivo, we generated *Pianp*-deficient (PianpKO) mice. PianpKO mice show neuroanatomical alterations including reduced thickness of the granule cell layer in the dentate gyrus (DG) and reduced numbers of Purkinje cells in the cerebellum. These alterations were accompanied by a significant deficit in presynaptic GBR-mediated inhibition of glutamate release, increased neuronal apoptosis, altered gene expression including reduced expression of ASD candidate gene *Erdr1* and autism-like behavior including enhanced anxiety, spatial learning deficits, repetitive behavior and severely impaired social interactions.

## Results

### *Pianp* is preferentially expressed by neurons and *Pianp*-deficient mice exhibit morphologic and cellular alterations in the hippocampus and cerebellum

**Fig. 1:**
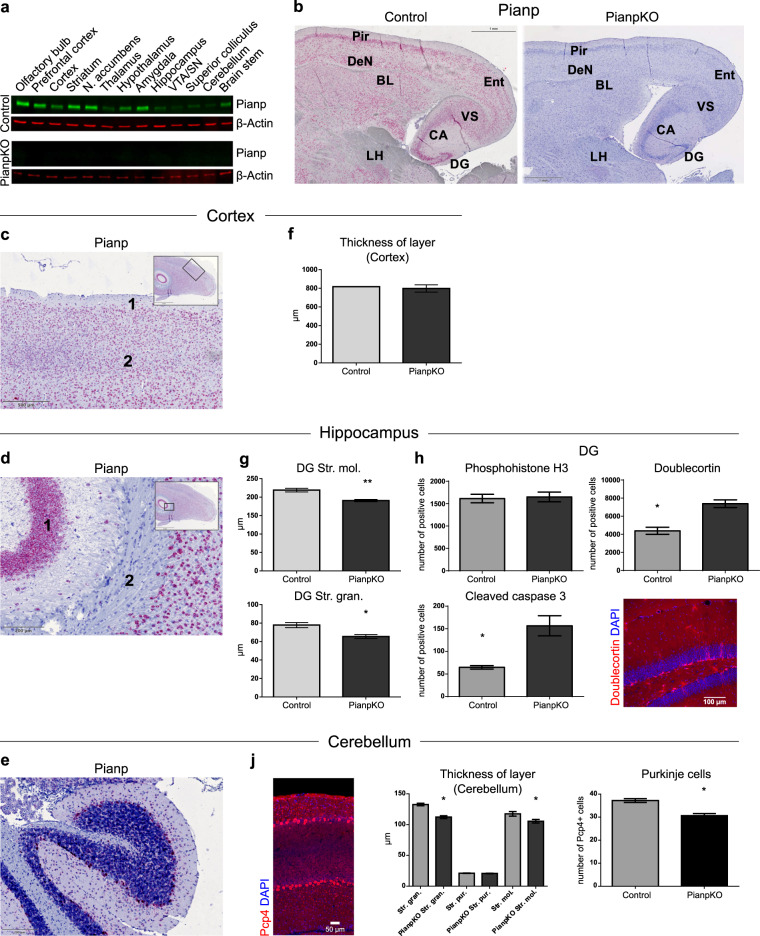
*Pianp* is predominantly expressed in neuronal cells and its deficiency leads to increased numbers of immature neuronal cells and enhanced apoptosis in the hippocampus as well as reduced numbers of Purkinje cells in the cerebellum. **a** Western blots of whole protein lysates isolated from different brain regions of control and PianpKO mice with anti-Pianp and anti-β-Actin antibodies. **b** ISH for *Pianp* in the CNS showing basolateral amygdala (BL), CA1–CA3 fields of the hippocampus (CA), dorsoendopiriform nucleus (DeN), dentate gyrus (DG), entorhinal cortex (Ent), lateral hypothalamus (LH), piriform cortex (Pir), and ventral subiculum (VS). *Pianp* expression (in red) was observed with a neuronal expression pattern in most brain regions of control but not PianpKO mice. **c** In the cortex layer I (1), which is mostly composed of astrocytes, only minor *Pianp* expression (in red) was detected by ISH in control mice, while the deeper layers II to VI of the cortex (2), which are mostly composed of neuronal cells, displayed strong *Pianp* expression. **d** *Pianp* expression (in red) was detected by ISH in control mice in hippocampal pyramidal neurons (1) but not in fiber tracts (2) of the hippocampus, in which predominantly glia cells occur. **e** In the cerebellum of control mice *Pianp* expression (in red) was predominantly observed in Purkinje cells by ISH. **f** The thickness of the primary somatosensory cortex did not significantly differ (*p* > 0.05) between PianpKO and control mice (*n* = 3 per group). **g** Assessment of layer thicknesses of the DG in PianpKO and control mice. Stratum moleculare (Str. mol.) (*t*(4) = 4.92, *p* < 0.01) and stratum granulosum (Str. gran.) (*t*(4) = 3.50, *p* < 0.05) were significantly thinner in PianpKO than in control mice (*n* = 3 per group). **h** Adult hippocampal neurogenesis. No significant difference (*p* = 0.6717) between PianpKO and controls was seen in the number of proliferating cells (phosphohistone H3-positive) in the DG of adult mice (*n* = 6 per group). However, the numbers of immature neuronal cells (doublecortin-positive, +68%, *p* = 0.0008) and apoptotic cells (cleaved caspase 3-positive, +142%, *p* = 0.0025) were significantly higher in PianpKO compared with control mice (*n* = 6 per group). **j** Assessment of layer thicknesses and cellular composition of the cerebellum of PianpKO and control mice (*n* = 3 per group). A significant reduction in the thickness was noted in PianpKO mice in the stratum granulosum (Str. gran.) (*t*(4) = 3.5, *p* < 0.05) and the stratum moleculare (Str. mol.) (*t*(4) = 4.92, *p* < 0.05). The thickness of the Stratum purkinjense (Str. pur.), which only contains a single row of soma of Purkinje cells, was not altered, however, the density of Purkinje cells was significantly reduced in PianpKO mice as compared with their controls (*t*(10) = 5.30, *p* < 0.05, *n* *=* 6 per group). Bars indicate mean ± sem, *p < 0.05, **p < 0.01, two-tailed unpaired *t* test, thickness of layer (cerebellum): one-way ANOVA followed by the Sidak’s multiple comparison test. All images are representative for *n* ≥ 3

Pianp was expressed in all brain regions indicating broad but variable expression throughout the whole brain (Fig. 1a, b, and Supplementary Fig. 1A). On cellular level, in situ hybridization (ISH) confirmed *Pianp* expression in cortical layers II–VI (Fig. 1c), in hippocampal pyramidal neurons, in granule cells of the DG (Fig. 1d), and, within the cerebellum, especially in Purkinje cells (Fig. 1e). The pattern of expression indicated mostly neuronal expression as the fiber tracts of the hippocampus (Fig. 1d) and the cortex layer I were mostly negative (Fig. 1c). PianpKO mice did not show any Pianp expression in the brain (Fig. 1a) and ISH of the brain and liver using a probe targeting the junction of exons 3 and 4 confirmed complete recombination and global inactivation (Fig. 1b and Supplementary Fig. 1B). PianpKO mice were viable, fertile, and survived at least 24 months. Grossly, these mice had no physical abnormalities. Blood plasma analyses (electrolytes, transaminases, cholinesterase, total protein, glucose, cholesterol, triglycerides, and urea) and routine staining of visceral organs did not reveal major abnormalities such as pathologic fibrosis or inflammation (Supplementary Figs. 2 and 3). The architecture and density of the vasculature of the liver appeared normal as shown by contrast-enhanced ultrasound (CEUS) and magnetic resonance imaging (MRI) (Supplementary Fig. 4). Mouse embryonic fibroblasts, that do not exhibit endogenous Pianp expression, were transfected with empty vector (EV) and *Pianp*. While proliferation was not altered, adhesion, and transwell-migration were increased in MEF-Pianp in comparison to MEF-EV indicating that Pianp can be involved in adhesion and migration (Supplementary Fig. 5).

The overall brain volume was slightly larger in PianpKO mice compared with control mice with a trend towards statistical significance (Supplementary Fig. 6A). MRI volume measurements revealed a significantly lower volume of the prefrontal cortex in the PianpKO group compared with the controls while no differences were detected in the volume of the striatum and hippocampus (Supplementary Fig. 6B). Global size and structure as well as functional parameters derived from diffusion-weighted MR imaging of the brain appeared unaltered (Supplementary Fig. 6C). As MRI is not as sensitive as morphometric histologic measurements, neuroanatomic analyses of several brain regions were performed. While the thicknesses of the neocortex (Fig. 1f), the corpus callosum-alveus, and the external capsule were not altered in PianpKO mice (Supplementary Fig. 7A), layering and cellularity of the hippocampus and cerebellum differed in comparison to control mice. In the hippocampus decreased thicknesses of the stratum granulosum and the stratum moleculare of the DG (Fig. 1g) correlated with enhanced apoptosis of granule cells indicated by an increase of cleaved caspase 3 staining in PianpKO mice (Fig. 1h). On the other hand, the thicknesses of CA1 subregions stratum oriens, stratum pyramidale, stratum radiatum, and stratum lacunosum were unaltered (Supplementary Fig. 7A). Although proliferating phosphohistone H3-positive cells did not differ significantly in the DG of adult PianpKO and control mice (Fig. 1h), there was a notable and significant increase in the number of doublecortin-positive cells in the PianpKO mice, indicating enhanced abundance of newly formed, immature neuronal cells (Fig. 1h).

Measurements within the cerebellum demonstrated a significant reduction in the thicknesses of the granule cell layer and the molecular layer in PianpKO mice (Fig. 1j). Although the thickness of the Purkinje cell layer was not significantly altered, the density of Purkinje cells was significantly reduced in PianpKO mice (Fig. 1j).

### Pianp localizes to axonal and dendritic neuronal processes and is involved in presynaptic GBR inhibition

**Fig. 2:**
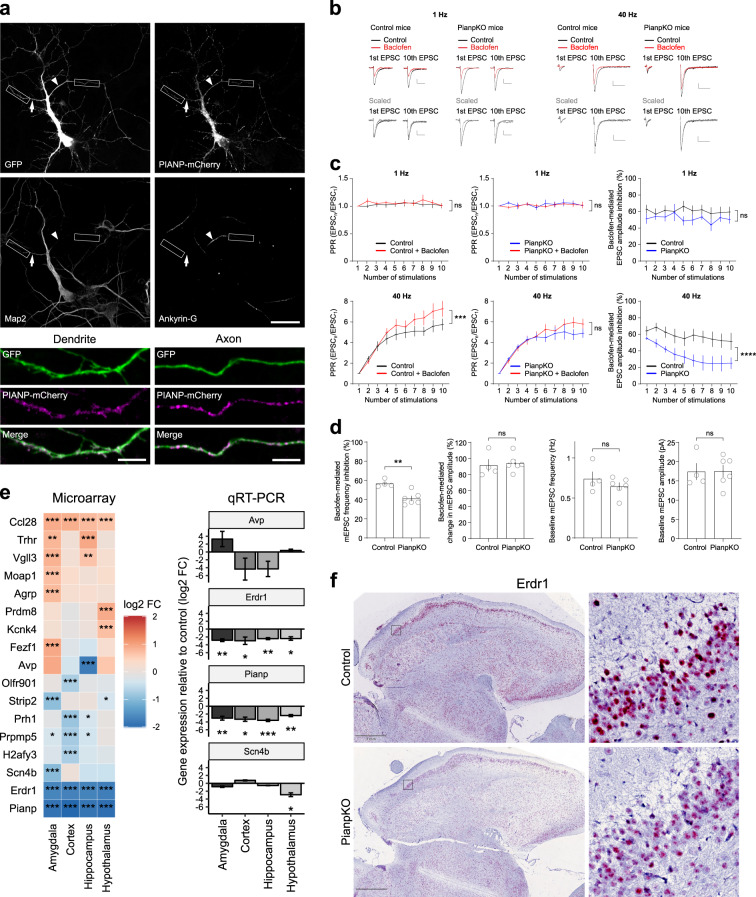
Pianp is sorted into axons and dendrites in neurons and controls presynaptic GBR inhibition as well as gene expression in the brain. **a** Overexpressed PIANP is sorted into axons and dendrites in cultured hippocampal neurons. At 7 days in vitro, PIANP-mCherry and GFP plasmids were transfected in wild-type hippocampal neurons and overexpressed for 6 h before fixation. GFP served as a volume marker. Representative co-transfected cell, demonstrating axonal and somatodendritic localization of PIANP-mCherry. Higher magnification images of axonal and dendritic segments correspond to insets in overview image. Map2 was used as a marker of dendrites (arrow) and Ankyrin-G was used as a marker of the axon initial segment (arrowhead). Scale bars: 30 μm (overview images), 5 μm (high-magnification images). **b**–**d** Reduced presynaptic GBR-mediated inhibition in PianpKO mice. **b** Sample traces of evoked EPSCs from CA1 pyramidal neurons of PianpKO and control mice. EPSCs were evoked by train stimulation at 1 Hz or 40 Hz in the absence (black traces) and in the presence of 100 µM baclofen (red). The 1st EPSC in the presence of baclofen is scaled to that in the absence of baclofen (gray traces) to compare paired pulse ratios (PPR) of the 1st to the 10th EPSC. Scale bars, 50 ms and 50 pA. **c** Train stimulation at 40 Hz reveals a significant deficit in presynaptic GBR inhibition in PianpKO mice. Control (left: eight cells, three mice) and PianpKO (middle: nine cells, four mice) EPSCs are scaled to the 1st EPSC (Control 1 Hz, *p* = 0.1737; PianpKO 1 Hz, *p* = 0.3836; Control 40 Hz, *p* = 0.0022; PianpKO 40 Hz, *p* = 0.0602). Baclofen-mediated EPSC amplitude reduction is significantly decreased in PianpKO mice during 40 Hz train stimulation (right: 1 Hz, *p* = 0.0705; 40 Hz, *p* < 0.0001). **d** Impaired GBR-mediated inhibition of the mEPSC frequency in CA1 pyramidal neurons PianpKO mice (*p* = 0.003). The mEPSC amplitude remained unchanged (*p* = 0.7576). Basal mEPSC frequency (*p* *=* 0.3304) and amplitude (*p* = 0.9653) were unaltered. Data are from four cells from two control mice and seven cells from three PianpKO mice. **e** The heat map of significantly dysregulated genes identified by microarray transcriptome analysis of RNA isolated from amygdala, cortex, hippocampus, and hypothalamus of control and PianpKO mice (*n* = 4 for amygdala of control mice, *n* = 5 for each other group). QRT-PCR of amygdala, cortex, hippocampus, and hypothalamus of control and PianpKO mice for *Avp*, *Erdr1*, *Pianp* and *Scn4b* (*n* = 5 for each group). Bars indicate gene expression relative to control in log2 fold change (FC) normalized to *Actb*. **f** ISH for *Erdr1* in the CNS. *Erdr1* (in red) is broadly expressed in the cerebrum of control mice (upper panel) and its expression is reduced in the cerebrum of PianpKO mice (lower panel). Scale bars indicate 1 mm. Images are representative for *n* ≥ 3. Bars indicate mean ± SEM. *p < 0.05, **p < 0.01, ***p < 0.001, ****p < 0.0001, **c** two-way ANOVA, **d**, **e** two-tailed unpaired *t*-test

To further elucidate neuronal Pianp functions, the distribution of ectopically expressed PIANP was assessed in cultured hippocampal neurons. PIANP-mCherry fusion was co-expressed with GFP for 6 h before cells were fixed and immunolabeled with neurite markers (Fig. 2a). In all cells analyzed, PIANP-mCherry was localized in axons and somatodendritic compartments. Using GFP as a volume marker, the analysis of the normalized axon/dendrite ratio of PIANP-mCherry revealed a slight tendency toward a preferential axonal localization (Supplementary Fig. 7B).

Therefore, presynaptic GBR inhibition at CA3-to-CA1 synapses of PianpKO mice was tested under conditions of repetitive stimulation. While presynaptic inhibition by baclofen in PianpKO mice was normal with 1 Hz train stimulation (Fig. 2b, c), PianpKO but not control mice revealed a significant deficit in baclofen-mediated inhibition of glutamate release with 40 Hz train stimulation (Fig. 2b, c), a paradigm that induces short-term facilitation because of presynaptic Ca^2+^ accumulation. With 40 Hz stimulation, baclofen significantly increased the paired-pulse ratio (PPR) in control mice but not in PianpKO mice (Fig. 2b, c), which therefore exhibit a faster saturation of facilitation. In line with a deficit in presynaptic GBR-mediated inhibition in PianpKO mice, we observed a significantly reduced baclofen-mediated inhibition of the miniature excitatory postsynaptic current (mEPSC) frequency, without a change in mEPSC amplitude (Fig. 2d). Baseline mEPSC frequency and amplitude in PianpKO mice were unaltered (Fig. 2d).

### Pianp regulates neuronal gene expression including autism-related gene *Erdr1*

Microarray gene expression analysis using whole-transcript arrays was performed with RNA isolated from the cortex, hippocampus, amygdala, and hypothalamus of control and PianpKO mice. Thereby, 17 genes were identified that were significantly regulated with a log2 fold change (FC) of >0.7 or <0.7 (corresponding to a FC of approx. >1.6 and <0.6) and *p* < 0.001 (Fig. 2e). As expected, *Pianp* was the gene with the strongest reduction in all regions, while only two other genes, *Ccl28* and *Erdr1*, were dysregulated significantly in all four regions. Genes that were regulated with a FC > 2 or <0.5 were also assessed by qRT-PCR. Significant downregulation of *Erdr1* was confirmed in all four regions (Fig. 2e). By ISH *Erdr1* displayed a neuronal expression pattern that was weaker throughout the brain in PianpKO mice in comparison to control mice indicating that *Pianp* deficiency led to a global decrease of *Erdr1* expression in neurons (Fig. 2f).

To evaluate whether these molecular alterations also affected neurotransmitter abundance, the monoamines 5-hydroxyindoleacetic acid (5-HIAA), serotonin (5-HT), dopamine (DA), 3,4-dihydroxyphenylacetic acid (DOPAC), homovanillic acid (HVA), and noradrenaline (NA) were measured in the prefrontal cortex, hippocampus, amygdala, nucleus accumbens, striatum, ventral tegmental area/substantia nigra, and dorsal raphe nucleus of control and PianpKO mice. DA levels were slightly decreased in the amygdala of PianpKO mice in comparison to control mice (trend to significance, *p* = 0.051) while levels of all other neurotransmitters did not differ between the groups in the other regions (Supplementary Fig. 8). These data indicate that Pianp is likely not involved in the direct regulation of these neurotransmitters.

### *Pianp*-deficient mice display endophenotypes associated with ASD

As the observed neuroanatomic alterations of the hippocampus and cerebellum and the impaired presynaptic GBR inhibition and altered neuronal expression pattern may impact on a wide range of neurological functions, PianpKO mice and control animals were subjected to a broad range of behavioral tests covering cognitive, affective, social, and motor domains. Motor functions were analyzed by grip strength assessment and the rotarod performance test. On the first experimental day, PianpKO mice showed a significantly higher grip strength and a higher latency to fall compared with the control mice (Supplementary Fig. 9A, B). As this difference could not be detected at the subsequent days, a motoric deficit seemed unlikely. In the startle and prepulse inhibition (PPI) test, a lower PPI was detected in PianpKO mice (Supplementary Fig. 9C). Post hoc analysis, however, revealed PPI deficits in PianpKO mice only at the prepulse levels 76 dB and 80 dB.

### Exploratory and anxiety-like behavior in *Pianp*-deficient mice

**Fig. 3:**
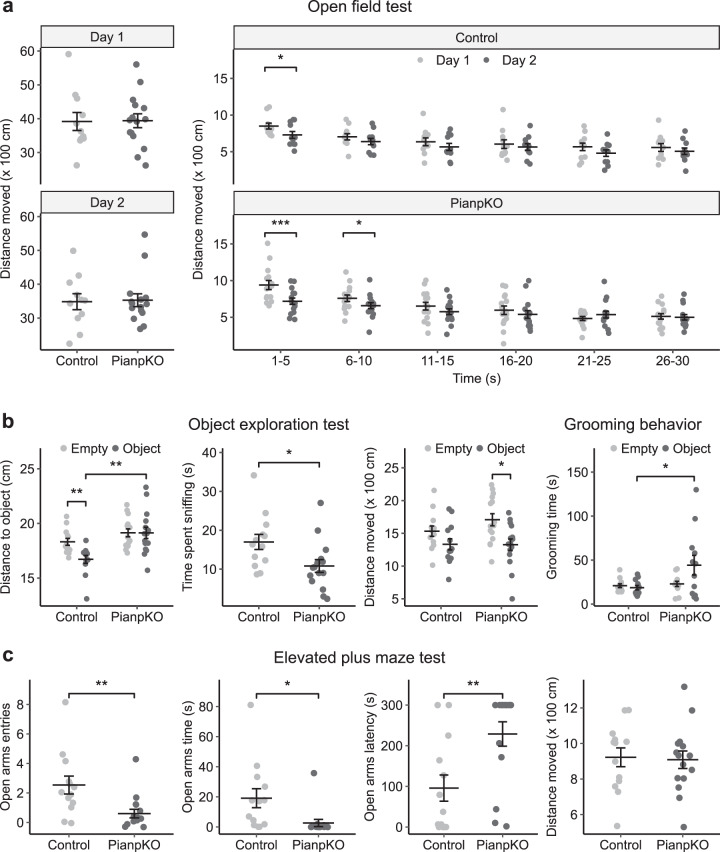
*Pianp*-deficient mice show increased anxiety in novel and aversive settings. **a** The total distance moved in the open field test (OFT) was not significantly different between control (*n* = 11) and PianpKO mice (*n* = 15) at day 1 (*t*(24) = 0.07, *p* > 0.05) or day 2 (*t*(24) = 0.14, *p* > 0.05). However, a higher distance moved in the first 5 min at day 1 compared with day 2 was detected in PianpKO (*t*(14) = 5.15, *p* > 0.001) as well as in control mice (*t*(10) = 2.83, *p* > 0.05). Between 6 and 10 min PianpKO mice still showed higher distance moved (*t*(14) = 2.16, *p* > 0.05), while this difference could not be detected in the control mice (*t*(10) = 1.68, *p* > 0.05). **b** In the object exploration test (OET), control mice (*n* = 13) showed significantly less distance to the position of the object when it was present (*t*(12) = 4.25, *p* < 0.01). PianpKO mice (*n* = 15) did not show this difference (*t*(14) = 0.01, *p* > 0.05) and had a significantly larger distance to the object than the control mice (*t*(26) = 3.70, *p* < 0.01). The time spent sniffing the object was significantly lower in PianpKO mice (*t*(26) = 2.45, *p* < 0.05). PianpKO mice moved significantly less when the object was present (*t*(14) = 2.58, *p* < 0.05) while this difference was not observed in control mice (*t*(12) = 1.73, *p* > 0.05). When the object was present, PianpKO mice (*n* = 12) showed a significantly longer time of grooming behavior compared with the controls (*n* = 12) (*t*(22) = 2.24, *p* < 0.05). **c** In the elevated plus maze test (EPM), PianpKO mice (*n* = 15) showed significantly less open arm entries (*t*(26) = 3.02, *p* < 0.01), a significantly shorter time spent on the open arms (*t*(26) = 2.57, *p* < 0.05), and a significantly higher latency to enter the open arms (*t*(26) = 3.03, *p* < 0.01) in comparison to control mice (*n* = 13). The total distance moved, however, did not differ significantly between the two groups (*t*(26) = 0.19, *p* > 0.05). Horizontal lines indicate mean ± SEM. *p < 0.05, **p < 0.01, ***p < 0.001, two-tailed unpaired or paired *t*-test

The open field test (OFT) assesses general exploratory behavior as well as general motor functions. In the open field the total distance moved by PianpKO mice did not significantly differ from the control mice, neither at the habituation day (day 1) nor at the following day (day 2). Comparison of the two experimental days, however, revealed a difference in the distribution of the exploratory drive. PianpKO mice showed a bigger reduction in the distance moved in the first 5 min between day 1 (habituation day) and day 2 (test day) in comparison to control mice. This difference was also still observed between 6 and 10 min in PianpKO while it was absent in control mice (Fig. 3a). However, the enhanced movement on day 1 was likely the result of an enhaced temporary stress response of PianpKO mice toward the novel environment. As general locomotor activity was unaltered, these findings indicate that the general exploratory behavior of PianpKO mice is normal which is important for the interpretation of further behavioral tests.

The object exploration test (OET) assesses the exploratory behavior related to an unfamiliar object. A significantly lower distance to the object position after presentation of the object was only detected in control but not in PianpKO mice. Control mice were exploring the object much more than PianpKO mice in terms of a significantly higher time spent sniffing and lower distance to the object position. Moreover, PianpKO mice showed a significantly lower distance moved after presentation of the unfamiliar object. Grooming time, an indicator of repetitive behavior, however, was significantly higher in PianpKO than in control mice after object presentation (Fig. 3b).

The elevated plus maze test (EPM) assesses the exploratory behavior in an insecure area. Here PianpKO mice explored the open arms less frequently, spent less time there, and showed a higher latency to the open arm exploration than control mice. The distance moved, however, did not differ between the groups (Fig. 3c).

The novelty-induced hypophagia (NIH) test showed only a trend towards higher latency to consumption and lower consumption in the novel cage in PianpKO mice as compared with control mice (Supplementary Fig. 9D). Overall, these tests indicate context-dependent enhanced anxiety-like behavior.

Amphetamine challenge was performed including a 30 min habituation phase and saline injection as control. As observed in other experimental setups, PianpKO moved longer distances compared with control mice only during the first ten minutes of the habituation phase but neither in the later phases of habituation nor after saline injection. Thirty minutes after amphetamine injection PianpKO started to show increased movement in comparison to controls, however not reaching statistical significance (Supplementary Fig. 9E).

### Stress response, emotionality, and autism-like behavior in *Pianp*-deficient mice

**Fig. 4:**
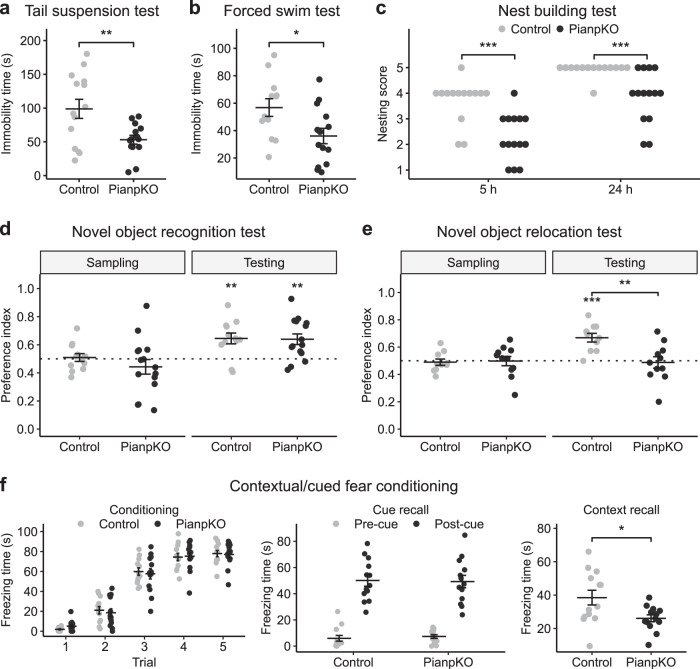
*Pianp*-deficient mice show an increased response to acute stress, a lower nesting score, no preference for a relocated object, and a deficit in contextual fear conditioning. **a** In the tail suspension test (TST), PianpKO mice (*n* = 14) moved more and had a significantly shorter time of immobility (*t*(26) = 2.93, *p* < 0.01) compared with control mice (*n* = 14). **b** In the forced swim test (FST), PianpKO mice (*n* = 14) swam for a longer time and had a significantly shorter time of immobility (*t*(24) = 2.44, *p* < 0.05) compared with control mice (*n* = 12). **c** In the nest building test (NBT), PianpKO mice (*n* = 14) had significantly lower nesting scores compared with the controls (*n* = 12) in the nest building after 5 h (*U* = 170.5, *p* < 0.001) and 24 h (*U* = 163, *p* < 0.001). **d** In the novel object recognition test (ORT), PianpKO (*n* = 15) (sampling: *t*(14) = 1.10, *p* > 0.05; testing: *t*(14) = 3.71, *p* < 0.01) and control mice (*n* = 12) (sampling: *t*(11) = 0.33, *p* > 0.05; testing: *t*(11) = 3.76, *p* < 0.01) showed a similar preference for the novel object that did not differ between the two groups (sampling: *t*(25) = 1.05, *p* > 0.05, testing: *t*(25) = 0.11, *p* > 0.05). **e** In the object relocation test (ORL), control mice (*n* = 10) showed a preference for the relocated object (sampling: *t*(9) = 0.45, *p* > 0.05; testing: *t*(9) = 5.30, *p* < 0.001) while PianpKO (*n* = 11) did not show a preference for the relocated object (sampling: *t*(10) = 0.06, *p* > 0.05; testing: *t*(10) = 0.29, *p* > 0.05). The preference index differed significantly between the two groups in the testing phase (sampling: *t*(19) = 0.20, *p* > 0.05; testing: *t*(19) = 3.38, *p* < 0.01). **f** In the contextual and cued fear conditioning, PianpKO mice (*n* = 12) and control mice (*n* = 13) showed a successful conditioning and comparable cue recall (*t*(24) = 0.13, *p* > 0.05). In context recall, however, PianpKO had a significantly lower freezing time (*t*(24) = 2.55, *p* < 0.05). Horizontal lines indicate mean ± SEM. *p < 0.05, **p < 0.01, ***p < 0.001, **a**, **b** two-tailed unpaired *t*-test, **c** Mann–Whitney *U* test, **d**, **e** one sample or two-tailed unpaired *t*-test

Tail suspension test (TST) and forced swim test (FST) were used to assess stress response [28, 29] and emotionality. In both tests PianpKO mice showed a significantly shorter time of immobility than control mice (Fig. 4a, b), indicating an increased stress response. The sucrose preference test did not show significant differences between the two groups (Supplementary Fig. 10A). Therefore, PianpKO mice did not exhibit features of a depression-like phenotype. In line with higher grooming times in OET (Fig. 3b), i.e. increased repetitive behavior, the nest building test (NBT) revealed significantly lower nesting scores in PianpKO mice compared with controls (Fig. 4c). These alterations suggest autism-like behavior.

### Cognition in *Pianp*-deficient mice

In the novel object recognition test (ORT) PianpKO and control mice both showed higher preference ratios for the novel object while exploration times did not significantly differ (Fig. 4d and Supplementary Fig. 10B). Although total exploration times were higher for PianpKO mice in both comparisons, in the object relocation test (OLT), however, only control mice showed a preference for the relocated object, while PianpKO mice did not show this preference (Fig. 4e and Supplementary Fig. 10C). Contextual and cued fear conditioning was performed successfully in PianpKO and control mice. Cue presentation resulted in similar freezing times. In context recall, however, PianpKO showed significantly lower freezing times (Fig. 4f). Taken together, PianpKO mice showed a specific deficit in spatial, but not nonspatial memory.

### Abnormal social behavior in *Pianp*-deficient mice

**Fig. 5:**
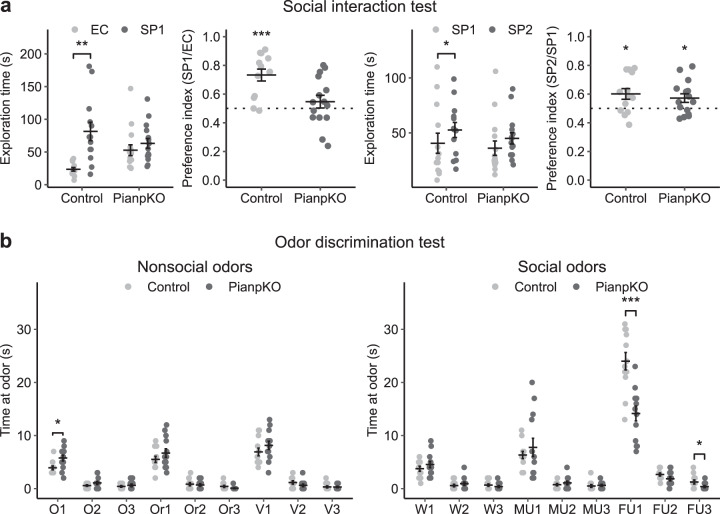
*Pianp* deficiency impairs social interaction and social odor discrimination. **a** In the social interaction test (SIT), PianpKO mice (*n* = 14) showed equal exploration times at an empty cage (EC) and at a cage with an unknown mouse (*t*(14) = 0.83, *p* > 0.05), designated as social partner mouse 1 (SP1), while the controls (*n* = 12), showed a significantly higher exploration time at SP1 than at the EC (*t*(12) = 4.08, *p* < 0.01). Therefore control mice had a statistically significant preference for SP1 (*t*(12) = 5.63, *p* < 0.001) that was not present in PianpKO mice (*t*(14) = 1.07, *p* > 0.05). Subsequent exposure to the familiar SP1 and to another unknown mouse, designated as social partner mouse 2 (SP2), PianpKO mice (*t*(14) = 2.38, *p* < 0.05) and controls (*t*(12) = 2.71, *p* < 0.05) both showed a slight, but statistically significant, preference for unknown SP2. However, the exploration times at SP1 and SP2 did only differ statistically significant in the control group (control: *t*(12) = 2.28, *p* < 0.05; PianpKO: *t*(14) = 1.58, *p* > 0.05). **b** In the odor discrimination test, PianpKO mice (*n* = 13) did not show a significant difference in the time spent sniffing the nonsocial odors oil (O2: *t*(23) = 1.49, *p* > 0.05; O3: *t*(23) = 0.69, *p* > 0.05), apart from the first round (O1: *t*(23) = 2.75, *p* < 0.05,), orange (Or1: *t*(23) = 1.18, *p* > 0.05; Or2: *t*(23) = 0.37, *p* > 0.05; Or3: *t*(23) = 1.68, *p* > 0.05), and vanilla (V1: *t*(23) = 1.16, *p* > 0.05; V2: *t*(23) = 1.38, *p* > 0.05; V3: *t*(23) = 0.91, *p* > 0.05) in comparison to the controls (*n* = 12). However, PianpKO mice spent a significantly shorter time sniffing female urine (FU1: *t*(23) = 4.59, *p* < 0.001; FU3: *t*(23) = 2.15, *p* < 0.05) compared with the controls (*n* = 12). However, FU2 did not differ significantly (FU2: *t*(23) = 1.73, *p* > 0.05). No significant difference between the groups could be detected for water (W1: *t*(23) = 1.03, *p* > 0.05; W2: *t*(23) = 0.87, *p* > 0.05; W3: *t*(23) = 0.99, *p* > 0.05) and male urine (MU1: *t*(23) = 0.76, *p* > 0.05; MU2: *t*(23) = 0.81, *p* > 0.05; MU3: *t*(23) = 0.36, *p* > 0.05). * *p* < 0.05, ** *p* < 0.01, *** *p* < 0.001, **a** one sample or two-tailed unpaired *t*-test, **b** two-tailed unpaired *t*-test

As similar cerebellar anomalies as found in PianpKO mice were also described in human patients with ASD [14] and murine models of the disease [15–17], social and repetitive autism-like behaviors were tested. In the social interaction test (SIT) control mice but not PianpKO mice showed a significantly different exploration time and preference for the social partner compared with the empty cage. Therefore, in contrast to control mice, PianpKO mice did not exhibit a normal preference for the chamber with a social partner. In comparing a known social partner (SP1) with a novel social partner (SP2) both PianpKO and control mice showed a preference for the novel social partner. However, the exploration times at SP1 and SP2 differed significantly only in control mice, indicating reduced interest of PianpKO mice in novel social interaction (Fig. 5a). In the odor discrimination test a general deficit in the discrimination of nonsocial odors could not be detected. The difference detected in the very first solvent control can likely be attributed to the increased stress response of PianpKO mice in novel settings. In the discrimination of social odors, PianpKO mice showed a significantly lower time sniffing at female urine, one of the strongest social cues, while sniffing time at water controls as well as at male urine did not significantly differ from control mice (Fig. 5b). Together, these results imply strong social impairment which in synopsis with the other results, i.e. repetitive behavior and enhanced anxiety, can be interpreted as autism-like behavior.

## Discussion

The global phenotype of PianpKO mice indicates that Pianp is primarily involved in the regulation of neuronal function and behavior as no major phenotypic or functional alterations were found in other organs beside the brain. Detailed phenotyping revealed that lack of Pianp affected a range of behavioral traits. Altogether, the results of OET and EPM demonstrated heightened anxiety in PianpKO mice. As the NIH test did not indicate heightened anxiety, modulation of anxiety by Pianp may be context dependent. The trend to lower DA concentrations in the amygdala of PianpKO may suggest that altered dopaminergic signaling could be involved as DA signaling in the amygdala has been described to affect anxiety [30–39]. Anxiety-like behavior is also present in mice with Purkinje cell-specific deficiency of *Shank2* [40]. Thus, enhanced anxiety in PianpKO could be mediated by altered output signaling from the cerebellum.

Results from the ORT and OLT reveal a specific deficit in spatial, but not nonspatial memory in PianpKO mice. The comparably richer set of cues available in the ORT (e.g. object shape, size, etc.) might be sufficient to provide the animal with enough information to recognize object novelty. With longer intervals (delays) between trials, however, a deficit in object novelty recognition might still become obvious. Hippocampus-independent auditory fear conditioning was normal, but PianpKO mice displayed significantly lower freezing in the hippocampus-dependent context recall. Taking these results together, a hippocampal deficit can be assumed in PianpKO mice. Regarding the hippocampus a decreased thickness of the stratum granulare and the stratum moleculare of the DG was observed. This is a result of altered adult neurogenesis and differentiation in this region. The number of doublecortin-positive, i.e. newly formed and immature neuronal cells was increased in PianpKO mice. However, also the rate of apoptotic cell death was strongly increased in PianpKO mice. These findings indicate altered differentiation of doublecortin-positive cells, which is likely delayed and may contribute to enhanced susceptibility to apoptosis. The observed imbalance of normal proliferation and enhanced apoptosis explains the net cell loss yielding a reduction in the thickness of the stratum granulosum and stratum moleculare of the DG. Using electrophysiology, a deficit in the baclofen-mediated inhibition of glutamate release was observed at CA3-to-CA1 synapses when recording mEPSCs or evoked EPSCs with 40 Hz train stimulation. The observed deficit in GBR-mediated presynaptic inhibition indicates that Pianp influences GBR regulation of synaptic transmission. The faster saturation of short-term synaptic plasticity observed with PianpKO mice likely influences information transfer in neuronal networks and contributes to the observed behavioral phenotypes. As GBR affects adult hippocampal neurogenesis [41] and is involved in learning and memory [22], it is likely that Pianp affects cellular composition and altered transmission in the hippocampus at least partially via interaction with GBR. As apoptosis can also be regulated by modulation of adhesion complexes [42, 43], alteration of these complexes by Pianp [8, 9] may also be involved in the observed phenotype.

PianpKO mice also displayed alterations in the composition of the cerebellum. The thickness of the stratum granulare and stratum moleculare as well as the density of Purkinje cells were significantly decreased in PianpKO mice. One of the main symptoms upon cerebellar dysfunction in humans is ataxia. PianpKO mice, however, did not show deficits in basic motor functions. In a mouse model of spinocerebellar ataxia these tests also revealed normal results [44]. Mice, as four-legged animals, might be less sensitive to cerebellar alterations in terms of motor functions. In addition, similar pathologic cerebellar features as observed in PianpKO are also seen in human patients with ASD [14] and murine models [15–17]. These murine models also exhibit normal basic motor functions and only showed alterations in complex tasks of motor learning [17].

PianpKO mice did not show the typical preference for social vs. nonsocial exploration normally observed in mice indicating severe social impairment. In line with this, their interest for the strongest social cue (female urine) was also lower in comparison to controls. Given that exploration times in the social recognition stage of the SIT were comparable to control mice, PianpKO mice seem to exhibit a lack of social interest particularly in choice situations, one of the key symptoms seen in ASD. The described behavior of the boy with homozygous nonsense variant in *PIANP* [12] also indicates a lack in social interaction. In PianpKO mice, autism-like behavior is also supported by the higher grooming time in OET and lower nesting scores found in NBT [45–47]. Downregulation of *Erdr1* as seen in all brain regions of PianpKO mice has also been described in another genetic model of neurodevelopmental disorder with resemblance to autism [48]. Therefore, *Erdr1* is a potential mediator of this phenotype in both models. Context-dependent enhanced anxiety and learning can also be impaired in human ASD as well as murine models [15–17]. Therefore, behavioral analyses and pathologic features of PianpKO mice strongly indicated an ASD-like phenotype.

Although, it was already shown that Pianp is a constituent of the GBRs in the CNS [19, 20], we here provide the first experimental evidence that Pianp indeed alters GBR signaling in vivo. Although GBRs have not been reported to be directly involved in ASD [22, 23], several reports show that activation of GBRs by GBR agonist baclofen may improve ASD symptoms [49]. In the BTRB and C58 mouse models of autism, baclofen improves social deficits and repetitive behavior [49]. In constitutive NMDAR hypofunction, baclofen rescues behavioral deficits [50], and in 16p11.2 deletion mice, baclofen reverses cognitive deficits and improves social interactions [51]. These findings indicate that constitutive hypoactivation of GBRs may context-dependently contribute to the manifestation of autism-like behavioral traits. As Purkinje cells are recognized as key cells mediating autism-like phenotypes in mice [14–17] and Purkinje cells represent the neuronal cell type with the highest levels of GBRs [25] as well as high expression of Pianp, it appears reasonable that *Pianp* deficiency associated cerebellar alterations and Purkinje cell dysfunction contribute to autism-like behavior.

Overall, the behavioral phenotype of PianpKO mice including context dependent enhanced anxiety, learning deficits, altered stress coping, and impaired social interaction in synopsis with the neuroanatomic cerebellar alterations indicates an ASD-like phenotype with strong resemblance to several Purkinje cell-dependent models of this disease [15–17]. As the case description of a boy with homozygous nonsense variant in *PIANP* showed global developmental delay including deficits in learning and social interaction, it appears likely that PIANP has conserved functions in mice and humans that involve global development, learning and social interaction. Therefore, these findings further strengthen the role of PIANP as an important mediator in these processes and identify *PIANP* as a novel candidate gene associated with ASD.

Future research to unravel how *PIANP* controls these diverse molecular and cellular alterations may further improve our understanding of the still enigmatic fields of ID as well as ASD and may therefore open new avenues for molecular diagnostic and therapeutic approaches.

## Material and methods

### Generation of *Pianp* knockout mice

Pianp^tm1a(KOMP)Wtsi^ C57BL/6N-A^tm1Brd^ mouse embryonic stem cells were obtained from the KOMP repository (No. CSD70665). After reconstitution these mice were crossed with B6N.Cg-Tg(ACTFLPe)9205Dym/CjDswJ mice (Jax No. 019100) to generate *Pianp* floxed mice. These mice were further bred with B6.C-Tg(CMV-cre)1Cgn/J mice (Jax No. 006054) to generate constitutive knockout mice, denoted as PianpKO. Western blotting, ISH, neuroanatomical, transcriptomic, and behavioral experiments were performed using male PianpKO and control (*Pianp* floxed) mice aged 8–48 weeks. For electrophysiology experiments P20-P27 B6-Pianp^em1Bet^ mice [20], denoted as PianpKO and control (wild-type littermates) mice were used.

### Brain microdissection

The whole brain was cut coronally using a cryostat (Leica Biosystems, Nussloch, Germany) and manually microdissected using brain punches. Paxinos’ and Franklin’s the Mouse Brain in Stereotaxic Coordinates [52] was used for identification of the different brain areas.

### Western blotting

Dissected brain region tissue was lysed using RIPA buffer (Sigma-Aldrich, St. Louis, MO, USA). SDS-PAGE and immunoblotting were carried out as described previously [8]. Images were acquired with the Odyssey CLx imaging system (LI-COR Biosciences, Lincoln, NE, USA) and quantified using ImageJ 1.48i [53]. Primary antibodies: anti-Pianp (clone 9C7) [10], anti-beta-Actin (No. A2103, Sigma-Aldrich). Secondary antibodies coupled with IRDye680RD and IRDye800CW (LI-COR).

### In situ hybridization (ISH)

ISH was conducted on coronal sections of formalin-fixed paraffin-embedded (FFPE) brains using RNAscope 2.5 HD and BaseScope assays in red [54] (Advanced Cell Diagnostics, Newark, CA, USA). The following probes were used for hybridization: BaseScope probe BA-Mm-*Pianp*-2EJ (No. 705741) and RNAscope probe Mm-*Erdr1* (No. 465101).

### Thickness of layers, adult hippocampal neurogenesis, and Purkinje cell density

Thirty micrometers thick coronal sections were made using a VT1000 vibratome (Leica Biosystems). Immunofluorescence stainings were prepared using the following primary antibodies: forebrain: anti-CNPase (No. PA5-19551, Thermo Fisher Scientific, Waltham, MA, USA), cerebellum: anti-Pcp4 (No. sc-74816, Santa Cruz Biotechnology, Dallas, TX, USA). The thickness of different brain structures was measured on a series of six consecutive sections starting at Bregma −1.94 mm. To assess adult hippocampal neurogenesis the following antibodies were used: anti-phosphohistone H3 (No. sc-8656-R, Santa Cruz Biotechnology), anti-doublecortin (No. sc-8066, Santa Cruz Biotechnology), anti-cleaved caspase 3 (No. AB3623, Millipore, Merck, Darmstadt, Germany). To assess Purkinje cell density a region-of-interest of 600 × 600 µm was superimposed on the 6th cerebellar lobule (starting at ~ Bregma −6.6 mm) and the number of Purkinje cell profiles was determined. See supplementary information for a detailed description.

### Immunocytochemistry and image analysis

Embryonic day 16.5 mouse hippocampi were dissected and dissociated. At 7 days in vitro, PIANP-mCherry and GFP plasmids were transfected in WT hippocampal neurons and overexpressed for 6 h before fixation. GFP served as a volume marker. See supplementary information for a detailed description.

### Electrophysiology

Three-hundred micrometers thick hippocampal slices were prepared with a VT1200S vibratome (Leica Biosystems) and were incubated for 15 min at 32 °C in ACSF containing (in mM): 126 NaCl, 26 NaHCO_3_, 2.5 KCl, 1.25 NaH_2_PO_4_, 2 CaCl_2_, 1 MgCl_2_, and 10 glucose. Slices were kept at room temperature until recording at 32 °C submerged in a recording chamber perfused with ACSF. CA1 pyramidal cells were visually identified using a 40x objective with a BX51WI microscope (Olympus, Tokyo, Japan). Cells were voltage-clamped at −60 mV with a Multiclamp700B amplifier (Molecular Devices, San José, CA, USA). Spontaneous mEPSCs were recorded in the presence of 0.2 μM tetrodotoxin and 100 μM picrotoxin. EPSCs were evoked with extracellular monopolar current pulses generated by a custom made isolated current stimulator and applied via a patch-pipette filled with ACSF and positioned to activate the Schaeffer collaterals. All recordings were filtered at 4-10 kHz and digitized at 10–20 kHz with a Digidata 1550B digitizer (Molecular Devices).

### Microarray transcriptome analysis

Total RNA was extracted from dissected brain region tissue using the RNeasy Mini Kit (Qiagen, Hilden, Germany). Gene expression profiling was performed using Affymetrix GeneChip Mouse Gene 2.0 ST Arrays (Thermo Fisher Scientific). A custom chip definition format version 21 with Entrez based gene definitions was used to annotate the arrays. The raw fluorescence intensity values were normalized applying quantile normalization. Differential gene expression was analyzed with ANOVA using JMP Genomics 13 (SAS Institute, Cary, NC, USA). A false positive rate of *α* = 0.05 with false discovery rate correction was taken as the level of significance. The raw and normalized data have been deposited in NCBI’s Gene Expression Omnibus and are accessible through GEO Series accession number GSE124791 (https://www.ncbi.nlm.nih.gov/geo/query/acc.cgi?acc=GSE124791).

### Quantitative reverse-transcription PCR (qRT-PCR)

RevertAid H-Minus M-MuLV transcriptase (Thermo Fisher Scientific) was used for reverse transcription. QRT-PCR was performed in Stratagene Mx3005P system (Agilent, Santa Clara, CA, USA) using SYBR Green PCR Master-Mix (Thermo Fisher Scientific). Relative gene expression in relation to reference gene (*Actb*) was calculated using the 2^−ΔΔ***CT***^ method. See supplementary information for primer sequences.

### Behavioral analysis

The experimental protocols used in this study complied with national and international ethical guidelines and were approved by the animal welfare commission of the Regierungspräsidium Karlsruhe (Karlsruhe, Germany). Housing conditions were as described previously [55]. All experiments were conducted during the dark period of the day, in the animals’ active phase. Animals were allocated to the experimental groups according to their genotype and labeled with numbers without information on the genotype. Therefore, data acquisition was performed in blindfold manner. Animals were excluded from the analysis if certain quality parameters defined for every experiment were not met indicating failure of this animal in the experiment. OFT, EPM, TST, FST, OET, ORT, OLT, NBT, and ODT were conducted as described previously [55–59]. See supplementary information for a detailed description.

### Social interaction test (SIT)

SIT was conducted according to Moy et al. [60]. Here, two phases, called sociability and preference for social novelty, were tested. In brief, in a three chamber test apparatus the test mouse was placed in the middle chamber. After a 5 min habituation phase, an unfamiliar male mouse (social partner 1) was placed into a wire cage in one of the side chambers, while the opposite side chamber contained an empty wire cage (sociability phase). In the second phase another unfamiliar male mouse (social partner 2) was placed into the empty wire cage to test preference for social novelty. See supplementary materials and methods for a detailed description.

### Statistical analysis

Statistical analyses were performed using R 3.4.2 [61], SigmaPlot 11 (Systat Software, San Jose, CA, USA), Prism 6 (GraphPad Software, La Jolla, CA, USA), or Excel 2010 (Microsoft, Redmond, WA, USA). Sample size determination was performed separately for each experiment according to experience from previous experiments using an alpha level of 0.05 and a beta level of 0.20. Results are reported as mean ± SEM. For statistical testing two-tailed unpaired or paired *t*-test, one sample *t*-test, ANOVA, and Mann–Whitney *U*-test were used. The appropriate statistical test was chosen according to the requirements of each test (e.g. normal distribution or equal variance). Results were considered significantly different if *p* < 0.05.

## Supplementary information

Supplementary Information
