# Supplementary material for: Pianp deficiency links GABAB receptor signaling and hippocampal and cerebellar neuronal cell composition to autism-like behavior
Source: Mol Psychiatry. 2019 Sep 11;25(11):2979–93. doi: 10.1038/s41380-019-0519-9 (PMC7577901; doi:10.1038/s41380-019-0519-9)
Supplement: Supplementary file 1 — Supplementary Information [file 41380_2019_519_MOESM1_ESM.pdf]

Supplementary Information

Supplementary Figures

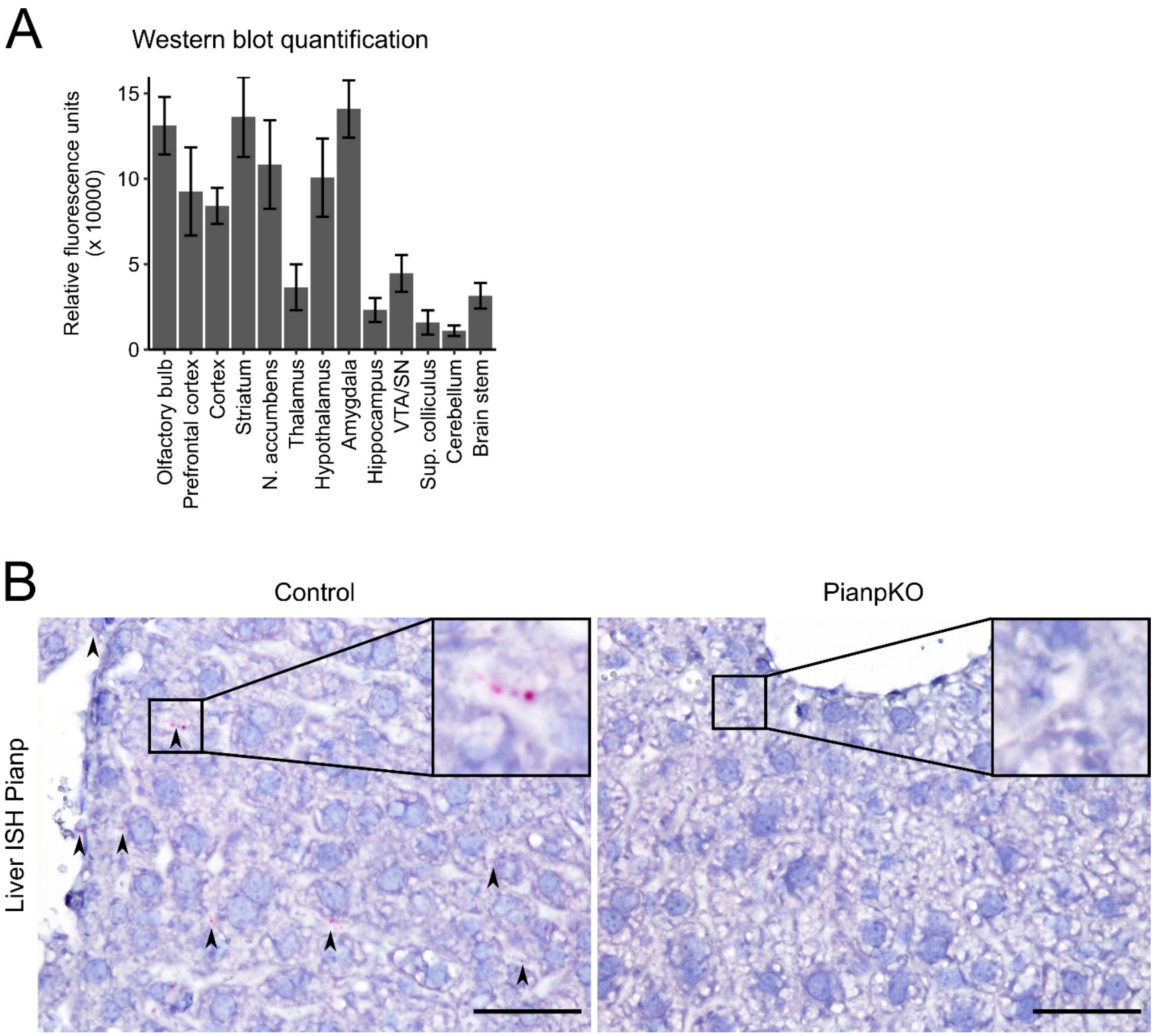

Supplementary Figure 1: Pianp expression in the brain and liver.

A) Quantification of the relative fluorescence of Pianp normalized to β-Actin in Western blots of different brain regions of control mice (n = 3). Bars indicate mean ± SEM.

B) In situ hybridization (ISH) for *Pianp* in the Liver. *Pianp* expression (in red, marked by black arrowheads) was observed in the liver predominantly in sinusoidal endothelial cells of control but not PianpKO mice. Scale bars: 50 µm. Images are representative for n ≥ 3.

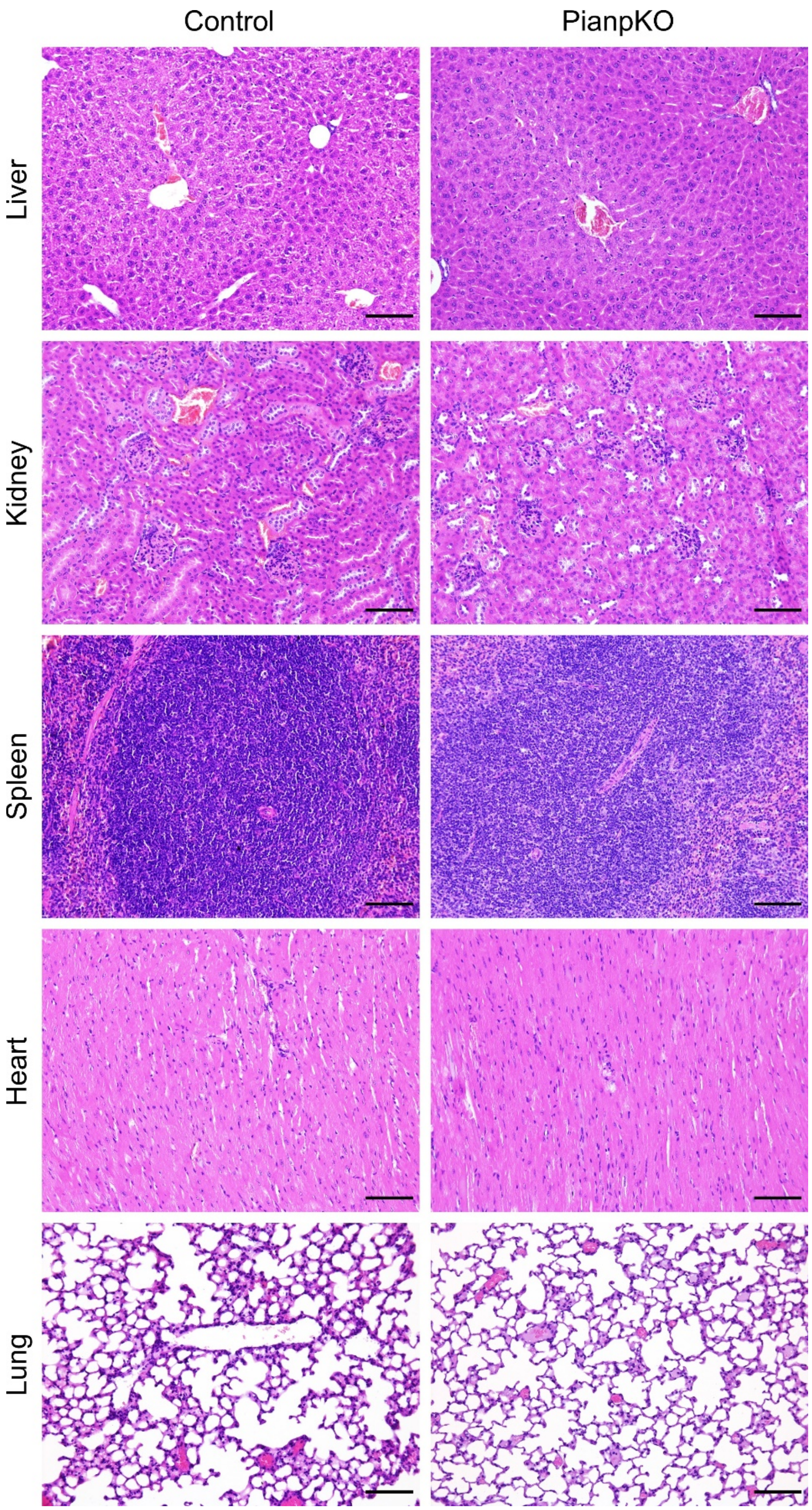

Supplementary Figure 2: H&E stains of the liver, kidney and spleen, heart, and lung revealed no obvious pathologies in PianpKO mice compared to controls. Scale bars: 100 µm. Images are representative for $n \geq 3$.

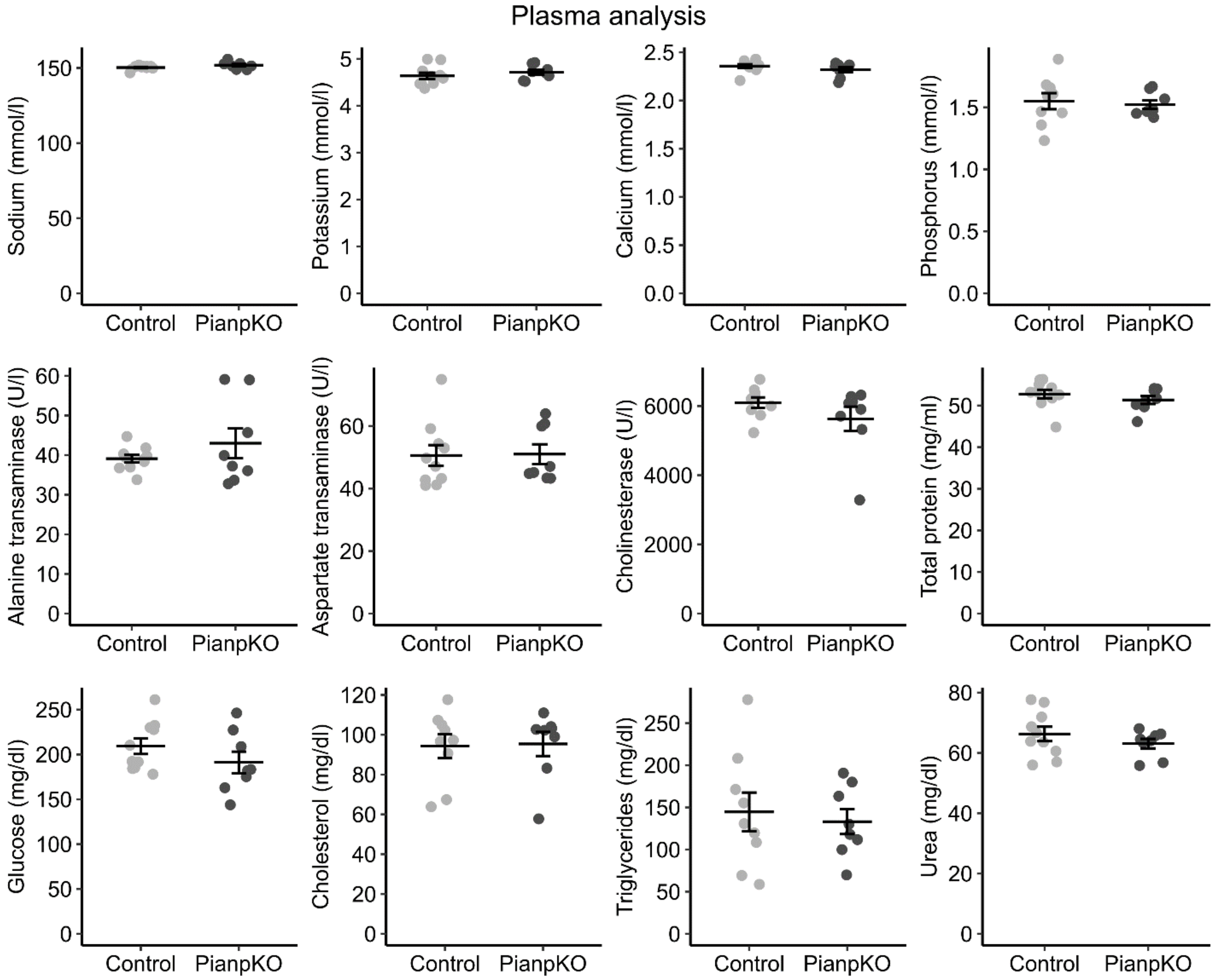

Supplementary Figure 3: Plasma analysis of blood samples taken from the ophthalmic venous sinus in PianpKO mice. No statistically significant differences between PianpKO mice and controls could be detected (*n* = 9 for each group). Horizontal lines indicate mean ± SEM.

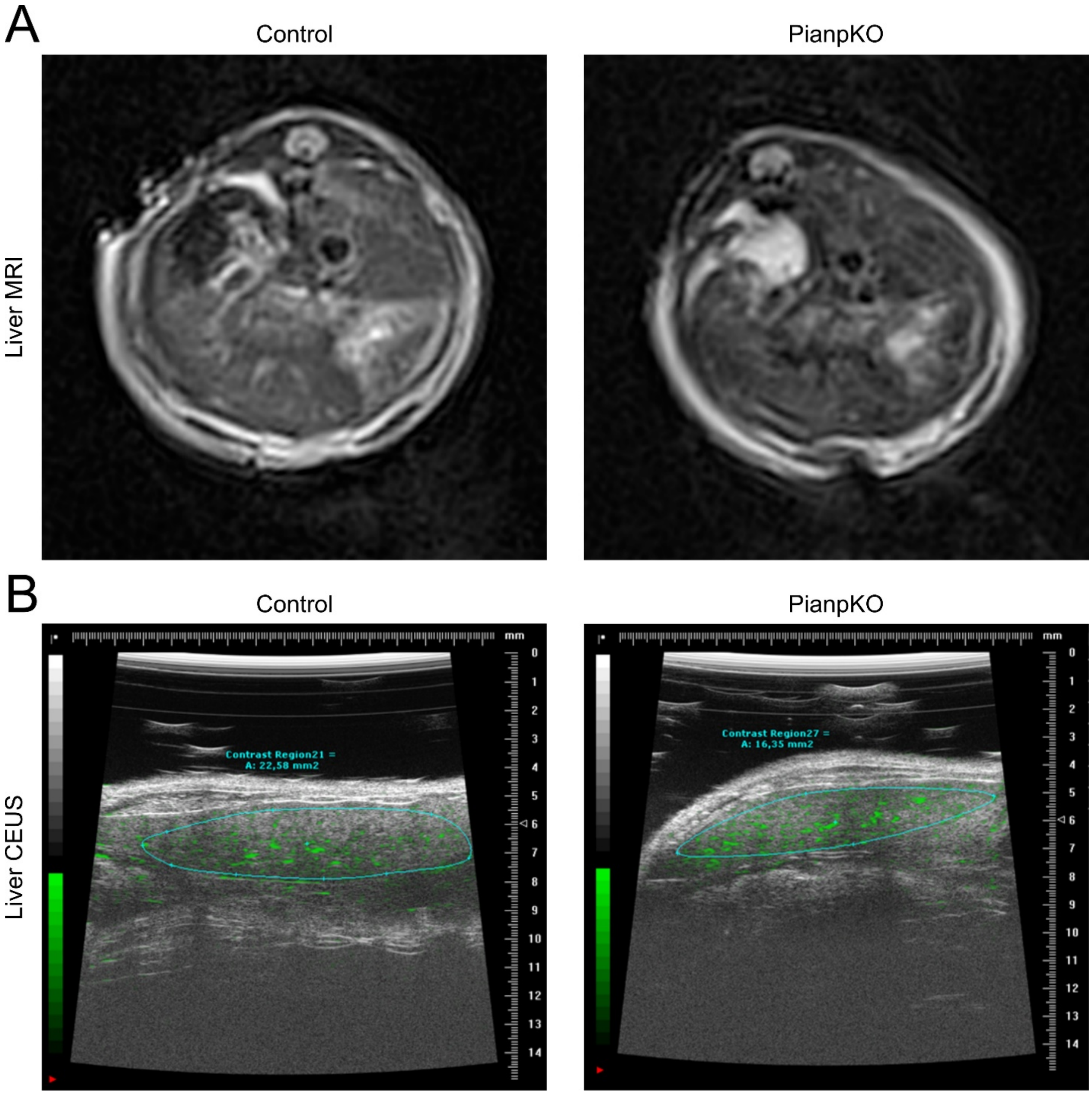

Supplementary Figure 4: Morphology and perfusion of the liver in Pianp-deficient mice.

A) 1.5 T magnetic resonance imaging (MRI) of the liver. No apparent morphological differences were detected between PianpKO mice and controls.

B) Contrast enhanced ultrasound (CEUS) imaging of the liver revealed no significant differences in liver perfusion between PianpKO mice and controls.

Images are representative for $n \geq 3$.

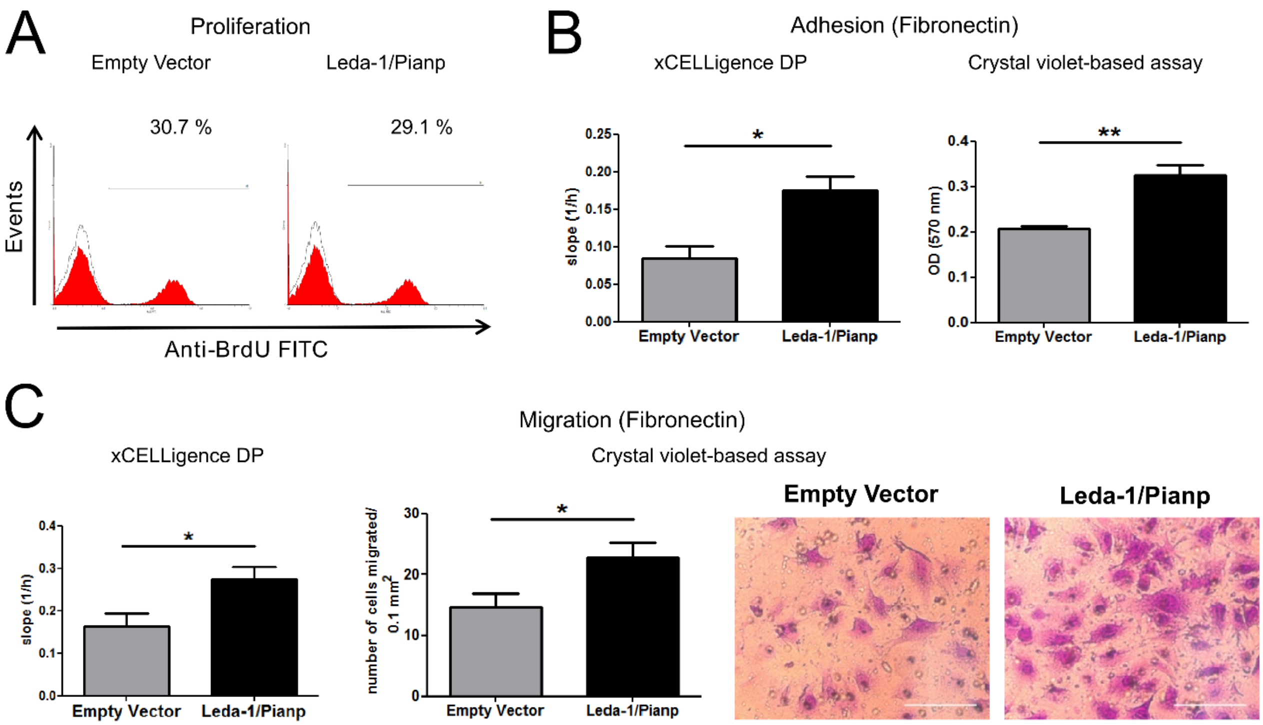

Supplementary Figure 5: Pianp overexpression in mouse embryonic fibroblasts (MEF)

A) Flow cytometry based BrdU assay. No differences in proliferation between empty vector and Pianp transfected MEF cells were detected.

B) Fibronectin coating adhesion assay. Pianp transfected MEF cells showed higher adhesion rates than empty vector transfected MEF cells in both xCELLigence DP and Crystal violet-based assay.

C) Fibronectin coated transwell migration assay. Pianp transfected MEF cells migrated significantly more through fibronectin coated transwell plates than empty vector transfected MEF cells in both xCELLigence DP and Crystal violet-based assay. Scale bar: 100 µm. Images are representative for $n \geq 3$.

Bars indicate mean ± SEM.

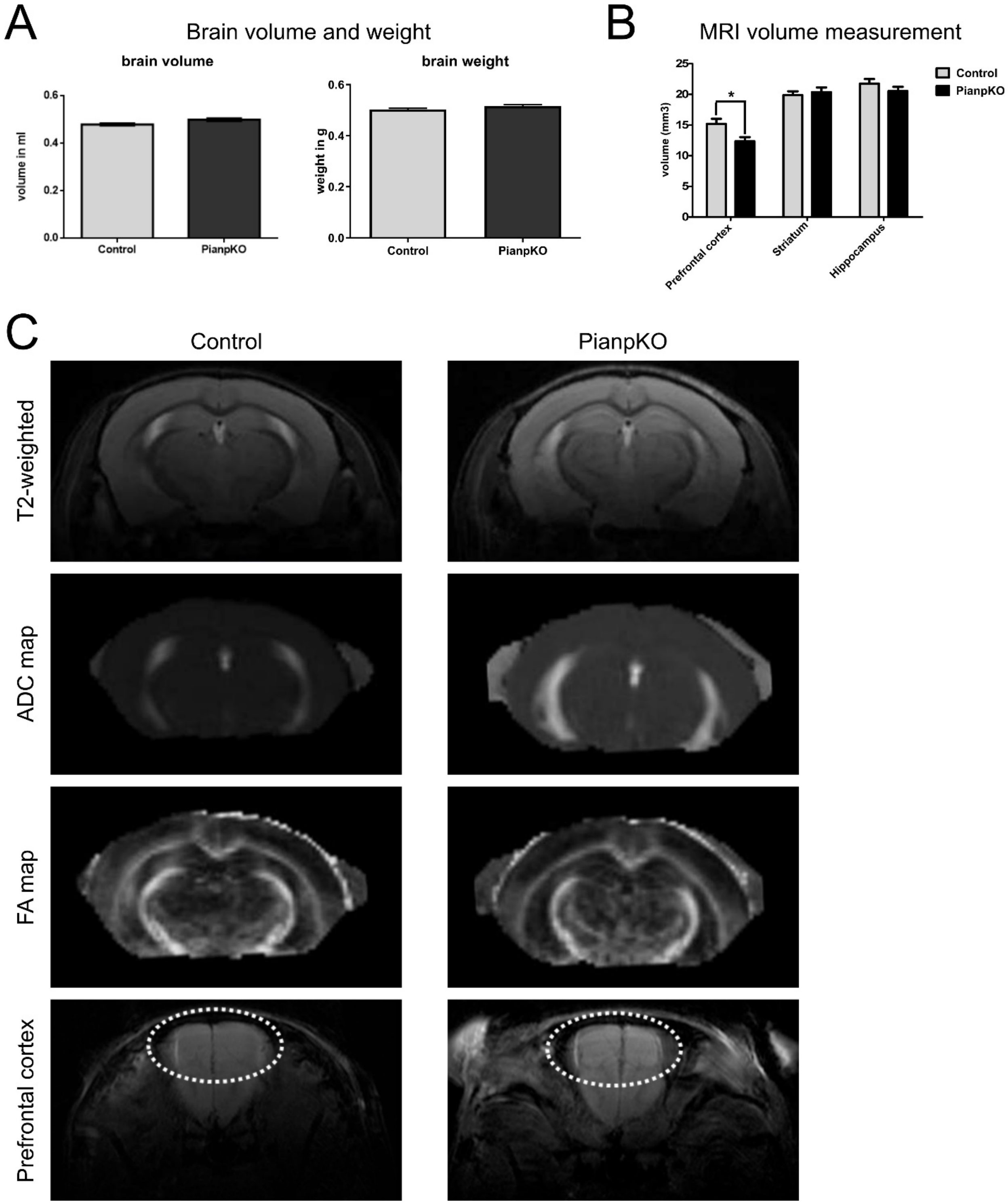
A
Brain volume and weight
brain volume
volume in ml
0.0
0.2
0.4
0.6
Control
PianpKO
brain weight
weight in g
0.0
0.2
0.4
0.6
Control
PianpKO
B
MRI volume measurement
volume (mm3)
0
5
10
15
20
25
*
Control
PianpKO
Prefrontal cortex
Striatum
Hippocampus
C
Control
PianpKO
T2-weighted
ADC map
FA map
Prefrontal cortex

Supplementary Figure 6: Volume, weight and anatomic and functional MRI of the brain in Pianp-deficient mice.

A) Brain volume of PianpKO mice was slightly increased, but did not reach statistical significance ($p = 0.0501$, Control: $n = 8$; PianpKO: $n = 9$). Brain weight of PianpKO mice did not differ statistically significant from controls ($p = 0.09$, Control: $n = 8$, PianpKO: $n = 9$).

B) Measurement of the volume of different brain regions of PianpKO and control mice using T2-weighted 9.4 T MR imaging. The volume of the prefrontal cortex differed statistically significant between PianpKO and control mice ($p = 0.015$, Control: $n = 9$; PianpKO: $n = 12$). However in striatum and hippocampus volume no statistically significant difference was detected ($p > 0.05$).

C) Morphological T2-weighted 9.4 T MRI as well as diffusion weighted imaging (which is made sensitive to water molecule diffusion in the brain) and diffusion tensor imaging (relying on the three-dimensional shape of the diffusion) of the brain in PianpKO and corresponding control mice. Representative images of coronal T2-weighted images of the hippocampal region (upper panel) and corresponding apparent diffusion coefficient (ADC) maps (middle upper panel) and fractional anisotropy (FA) maps (middle lower panel) of PianpKO and control mouse brains (week 61). Additionally, representative images of the prefrontal regions for both groups (lower panel). No statistically significant differences in parameters derived from DWI and DTI were noted when comparing PianpKO and control mice ($p > 0.05$, Control: $n = 9$; PianpKO: $n = 12$).

Bars indicate mean ± SEM.

A

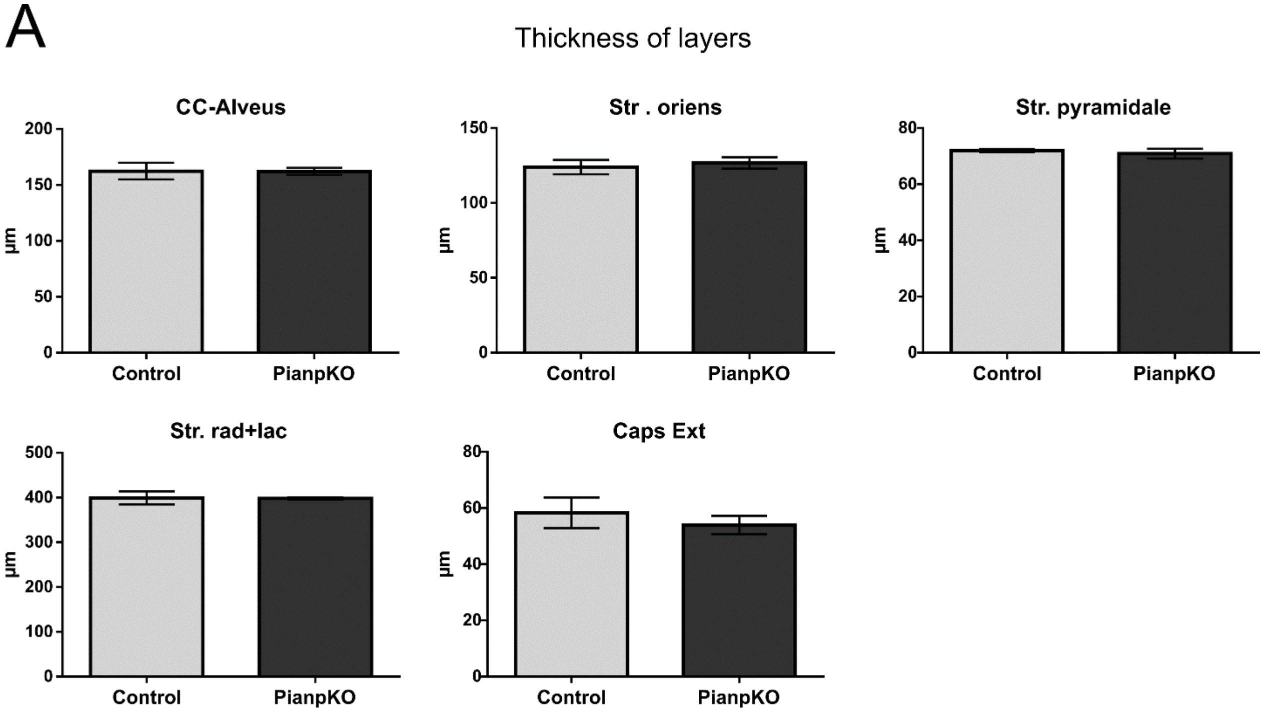

B

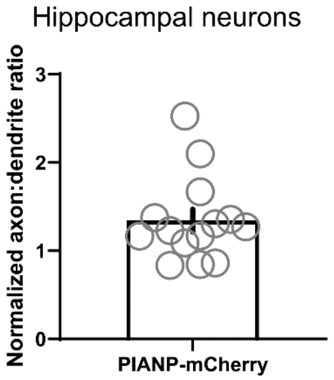

Supplementary Figure 7: Thickness of layers of fiber tracts in PianpKO mice and subcellular location of Pianp in hippocampal neurons.

A) PianpKO (*n* = 3) and control mice (*n* = 3) showed no differences in the layer width of the corpus callosum and alveus (CC-alveus), the stratum (str.) oriens, str. pyramidale, and str. radiatum (rad) plus lacunosum (lac) of the CA1 region of the hippocampus, and the external capsule (caps ext).

B) Quantification of PIANP-mCherry axon/dendrite ratio, normalized to GFP fluorescence in hippocampal neurons (*n* = 14 cells).

Bars indicate mean ± SEM.

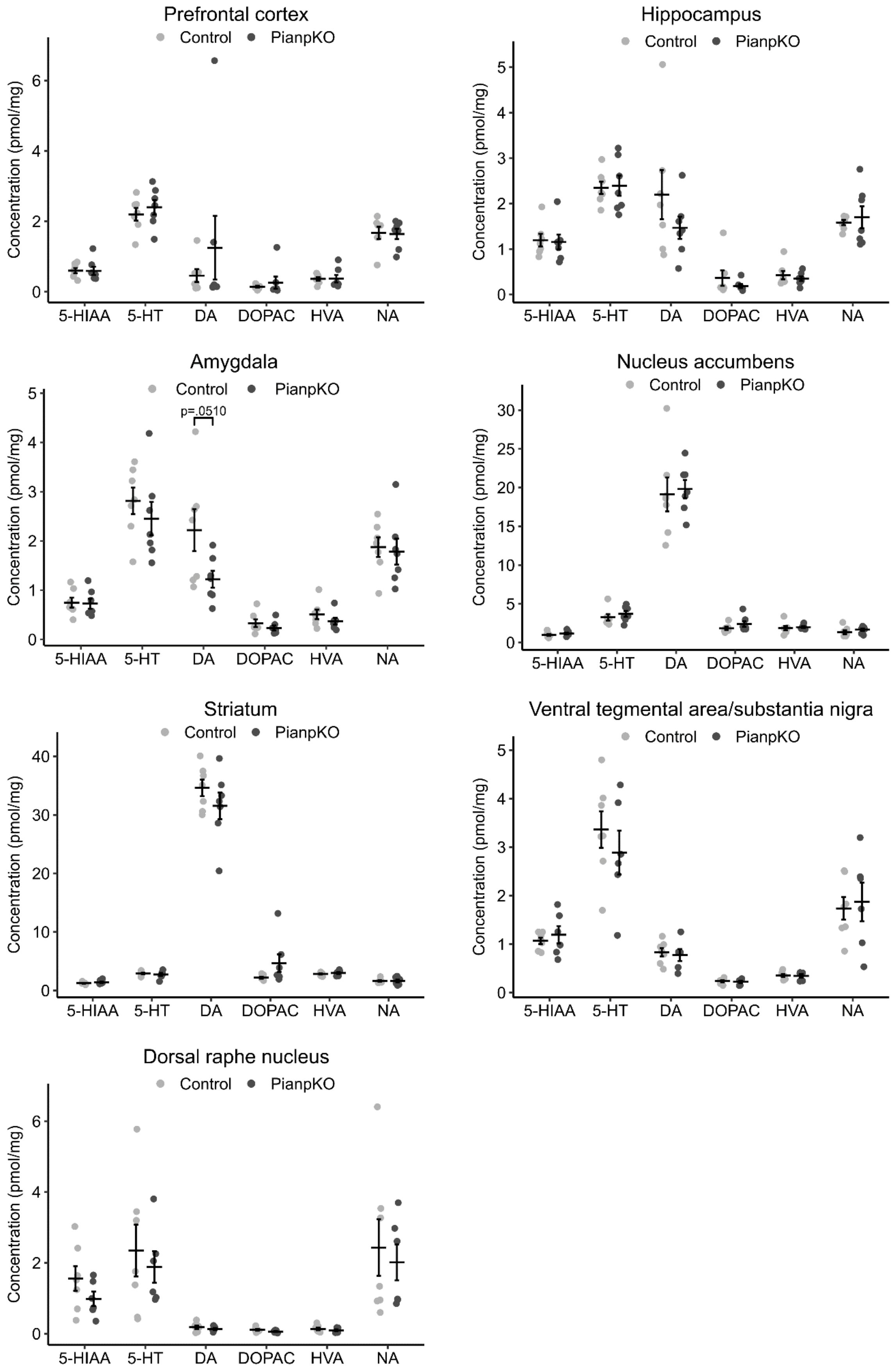
Prefrontal cortex
Control PianpKO
Concentration (pmol/mg)
5-HIAA 5-HT DA DOPAC HVA NA
Hippocampus
Control PianpKO
Concentration (pmol/mg)
5-HIAA 5-HT DA DOPAC HVA NA
Amygdala
Control PianpKO
p=.0510
Concentration (pmol/mg)
5-HIAA 5-HT DA DOPAC HVA NA
Nucleus accumbens
Control PianpKO
Concentration (pmol/mg)
5-HIAA 5-HT DA DOPAC HVA NA
Striatum
Control PianpKO
Concentration (pmol/mg)
5-HIAA 5-HT DA DOPAC HVA NA
Ventral tegmental area/substantia nigra
Control PianpKO
Concentration (pmol/mg)
5-HIAA 5-HT DA DOPAC HVA NA
Dorsal raphe nucleus
Control PianpKO
Concentration (pmol/mg)
5-HIAA 5-HT DA DOPAC HVA NA

Supplementary Figure 8: Impact of Pianp on monoamine neurotransmitter concentration in the brain. High-performance liquid chromatography (HPLC) measurement of monoamine neurotransmitters in different brain regions. 5-hydroxyindoleacetic acid (5-HIAA), serotonin (5-HT), dopamine (DA), 3,4-dihydroxyphenylacetic acid (DOPAC), homovanillic acid (HVA), and noradrenaline (NA) did not significantly differ between control and PianpKO mice in the prefrontal cortex, the hippocampus, the amygdala, the nucleus accumbens, the striatum, the ventral tegmental area/substantia nigra and the dorsal raphe nucleus. However, a trend ($t(12) = 2.17$, $p = 0.051$) towards reduced levels of Dopamine (DA) was observed in the Amygdala in PianpKO mice ($n = 7$ for each group). Horizontal lines indicate mean ± SEM.

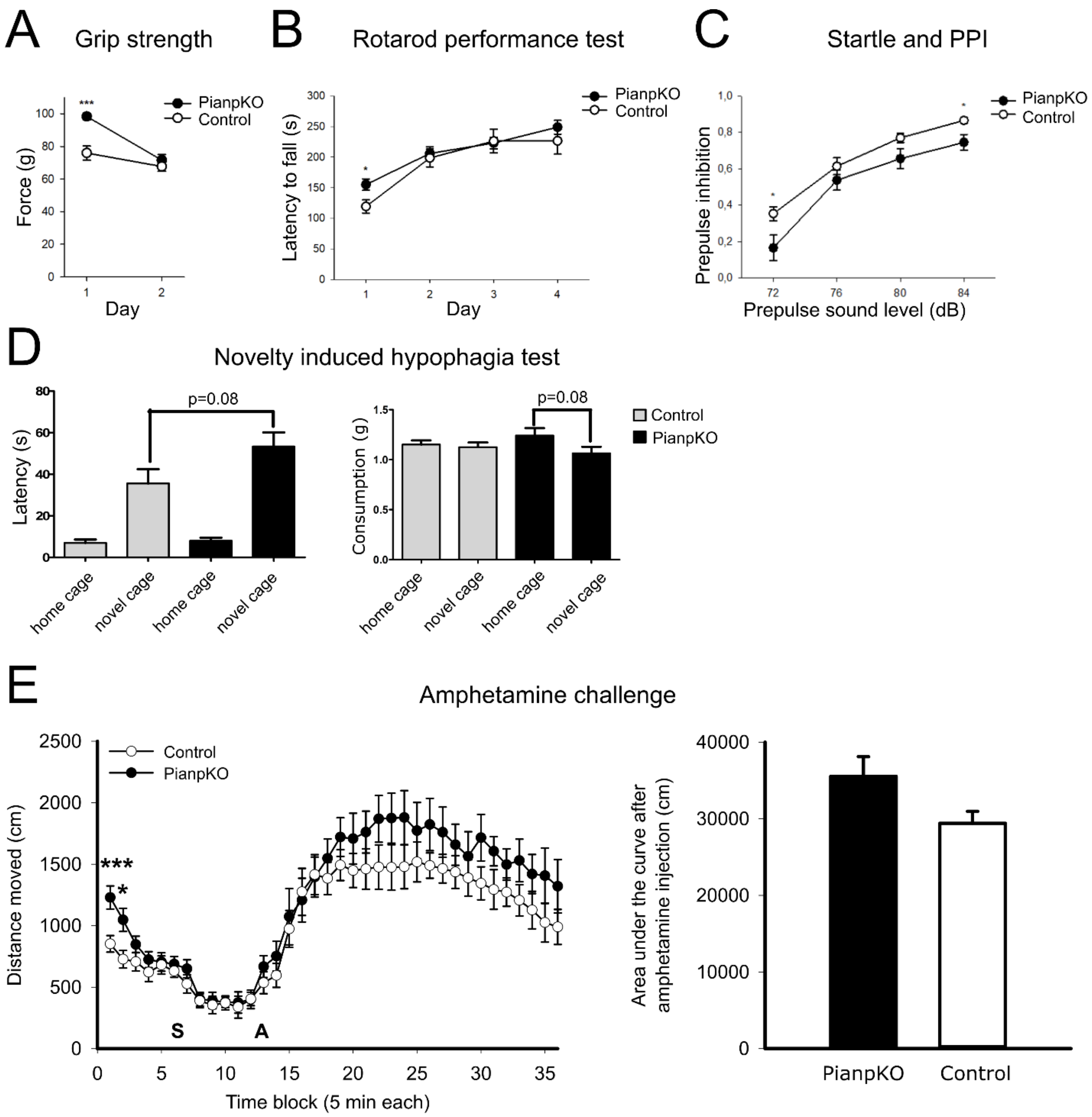
A
Grip strength
***
PianpKO
Control
Force (g)
Day
B
Rotarod performance test
*
PianpKO
Control
Latency to fall (s)
Day
C
Startle and PPI
*
*
PianpKO
Control
Prepulse inhibition
Prepulse sound level (dB)
D
Novelty induced hypophagia test
p=0.08
Latency (s)
home cage
novel cage
home cage
novel cage
p=0.08
Consumption (g)
home cage
novel cage
home cage
novel cage
Control
PianpKO
E
Amphetamine challenge
Control
PianpKO
***
*
S
A
Distance moved (cm)
Time block (5 min each)
Area under the curve after amphetamine injection (cm)
PianpKO
Control

Supplementary Figure 9: Motor functions, startle response/prepulse inhibition, anxiety behavior and amphetamine challenge in Pianp-deficient mice.

A) At the experimental day 1 grip strength was significantly higher in PianpKO (n = 15) than in control mice (n = 13) ($t(26) = 4.87$, $p < 0.05$). However on the following day 2, grip strength didn't differ significantly between the groups ($t(26) = 0.40$, $p > 0.05$).

B) At the experimental day 1 of the rotarod performance test, PianpKO mice (n = 15) showed a significantly higher latency to fall than control mice (n = 13) ($t(26) = 2.52$, $p < 0.05$). However on the following days 2 to 4 this difference was no longer observable (day 2: $t(26) = 0.68$, $p > 0.05$, day 3: $t(26) = 0.49$, $p > 0.05$, day 4: $t(26) = 0.35$, $p > 0.05$).

C) In the startle and prepulse inhibition (PPI) test, PianpKO mice (n = 15) showed a significantly lower PPI in comparison to control mice (n = 13, two way ANOVA: genotype $F(1,78) = 5.16$, $p < 0.05$, intensity $F(3,78) = 103.6$, $p < 0.0001$, genotype x intensity $F(3,78) = 0.98$, $p > 0.05$). Post hoc t-Tests however only revealed deficits at a sound level of 72 dB ($t(26) = 2.23$, $p < 0.05$) and 84°dB ($t(26) = 2.66$, $p < 0.05$) while 76 dB ($t(26) = 0.96$, $p > 0.05$) and 80 dB ($t(26) = 1.95$, $p > 0.05$) did not differ significantly between the two groups.

D) In the novelty induced hypophagia test, PianpKO mice (n = 14) showed a trend towards a higher latency to consumption in the novel cage in comparison to control mice (n = 11) ($t(23) = 1.82$, $p = 0.08$), and a trend towards a lower consumption in the novel cage compared to the home cage ($t(26) = 1.80$, $p = 0.08$).

E) Amphetamine challenge test with a 30 min habituation phase (time blocks 1–6 (5 min each)), a 30 min injection control phase (time blocks 7–12, S = saline injection) followed by the amphetamine challenge (time blocks 14–36, A = amphetamine injection). In the habituation phase differences could be detected in the first ($t(20) = 3.48$, $p < 0.001$) and second ($t(20) = 2.81$, $p < 0.05$) 5 min time blocks. In the amphetamine challenge test, PianpKO mice (n = 11) showed a trend towards a higher distance moved after amphetamine injection in comparison to control mice (n = 11) (two way repeated measures ANOVA: genotype $F(1,460) = 3.87$, $p = 0.063$, time $F(23,460) = 8.59$, $p < 0.001$, genotype x time $F(23,460) = 0.39$, $p > 0.05$; area under the curve: $t(20) = 2.04$, $p = 0.055$).

Dots/bars/horizontal lines indicate mean ± SEM. * $p < 0.05$, *** $p < 0.001$, A, B, D) two-tailed unpaired or paired t-Test, C) two way ANOVA followed by post hoc t-Tests

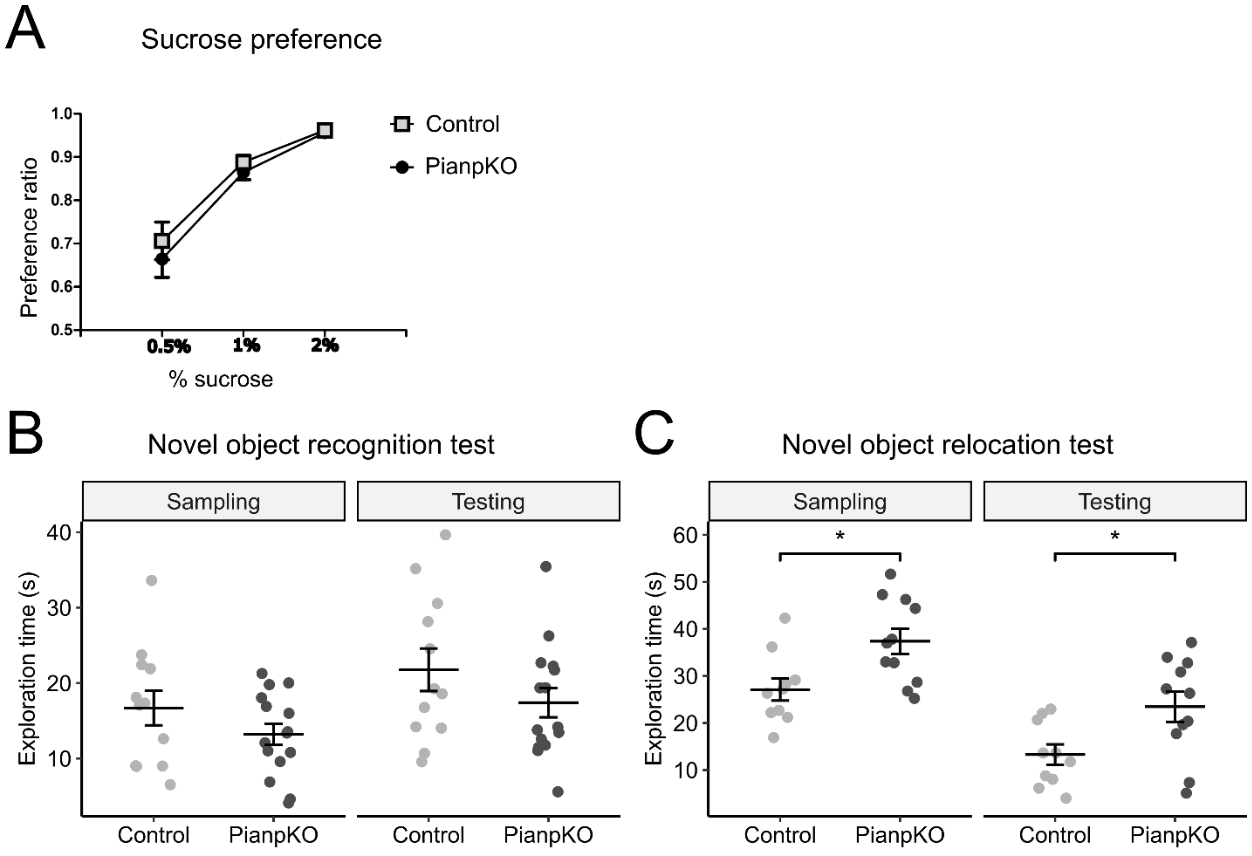

Supplementary Figure 10: Anhedonia and exploration time in novel object recognition and relocation tests in Pianp-deficient mice.

A) In the sucrose preference test for anhedonia no significant differences could be observed between PianpKO mice (n = 15) and control mice (n = 13) (0.5 % sucrose: $t(20) = 0.69$, $p > 0.05$, 1 % sucrose: $t(20) = 1.01$, $p > 0.05$, 2 % sucrose: $t(20) = 0.66$, $p > 0.05$).

B) In the novel object recognition test, the exploration time did not differ significantly between the groups (sampling: $t(25) = 1.35$, $p > 0.05$, testing: $t(25) = 1.31$, $p > 0.05$).

C) In the novel object relocation test, the exploration time differed significantly between the groups in both phases (sampling: $t(19) = 2.86$, $p < 0.05$, testing: $t(19) = 2.58$, $p < 0.05$).

Dots/bars indicate mean ± SEM, * $p < 0.05$, two-tailed unpaired or paired t-Test.

Supplementary Material and Methods

Organ Histology

Organs were fixed in 10 % formalin (Carl Roth, Karlsruhe, Germany) at room temperature for 24 to 72 hours, followed by paraffin embedding according to standard protocols. Paraffin sections (3 to 5 µm) were deparaffinized, rehydrated and H&E stained according to standard protocols.

Blood Plasma Analysis

Blood samples were taken from the ophthalmic venous sinus. Plasma was separated (centrifugation at 2,000 g for 5 minutes at room temperature in Microvette 500 LH; Sarstedt, Nümbrecht, Germany) and analyzed for sodium, potassium, calcium, phosphorus, alanine aminotransferase (ALT), aspartate aminotransferase (AST), cholinesterase, total protein, glucose, triglycerides, cholesterol, and urea in a cobas c 311 Analyzer (F. Hoffmann-La Roche, Basel, Switzerland). Calibration and independent controls were used as recommended by the manufacturer.

Liver MRI

Animals were anesthetized with the mixture of isoflurane (1.5 %) and room air. T2-weighted imaging was performed using clinical 1.5 T MR scanner (Symphony, Siemens, Germany) equipped with a home-built coil for radiofrequency excitation and detection, using following parameters: turbo spin echo sequence, orientation axial, TR 3240 ms, TE 81 ms, resolution 0.4×0.4×1.5 mm, 3 averages, 15 images, scan time 3:40 min. MR images were post-processed using OsiriX software (Pixmeo, Bernex, Switzerland).

Liver CEUS

Animals were anesthetized with the mixture of isoflurane (1.5 %) and room air. Contrast-enhanced ultrasound imaging was performed on a pre-clinical ultrasound platform Vevo 770 (Fujifilm VisualSonics, Toronto, Canada) using a RMV-704 probe with a center frequency of 40 MHz. After the start of the acquisition, SonoVue ultrasound contrast agent (Bracco, Amsterdam, Netherlands) was injected through a tail vein (approx. 100 µl in 10 seconds). Vevo-LAB software (Fujifilm VisualSonics) was used for the post-processing of ultrasound data according to the instruction of the manufacturer.

Brain Volume and Weight Measurement

Brain volume was determined by using microvolumetry (µ-VM) as described previously[1]. In brief, a 5-ml syringe (probe container) was attached to a 1-ml syringe (measurement devise). 70 % ethanol was used as fluid. First, the fluid level was set to a specific marker on the probe container. Thereafter, the probe was put into the probe container. Finally, the 1-ml syringe was used to set the fluid level in the probe container back to its initial value.

Brain MRI

Animals were anesthetized by inhalation of the mixture of isoflurane (2.0 %) and room air and temperature held constant at 36°± 0.5°C using water heated cradle. Mice were placed in a prone position on a plate holder with an adjustable nose cone. A stereotactic device was utilized together with respiratory gating to minimize head movement. *In vivo* imaging was performed on a 9.4 T Biospec 94/20 USR (Bruker, Karlsruhe, Germany) small animal imaging system. T2-weighted images and diffusion tensor images were acquired using a cryogenic surface coil (transceiver, quadrature) and a dual resonator system composed of a transmit-only volume resonator and a receive-only surface coil at room temperature (Bruker). For T2-weighted (RARE) images, imaging parameters were set to: TE/TR 35/4000 ms, matrix 256×256, field of view 20×20 mm, slice thickness 0.5 mm, 2 averages, scan time 4:16 min. For DTI-EPI, imaging parameters were: TE/TR 22/7500 ms, matrix 128×128, gradient separations time 8.5 ms, flip angle 90 degrees, slice thickness 0.5 mm, 1 average, 30 diffusion directions with b = 1000 s/mm^2 and 5 unweighted b=0 s/mm^2 images, acquisition time 17:30 min. T2-weighted MR images were segmented using OsiriX software (Pixmeo). Segmentation was performed manually slice by slice, in the coronal plane view. Three regions of interest (prefrontal cortex, caudoputamen, and hippocampus) were outlined manually. The boundaries of these three structures were chosen by visually comparing the greyscale images to three online mouse atlases: MBL mouse brain atlas[2], CFA mouse brain atlas[3] and scalable Brain Atlas[4]. After segmentation, the volume was calculated based on the number of segmented voxels, for prefrontal cortex, caudoputamen, and hippocampus, respectively. The diffusion tensor imaging was analyzed using DSI Studio (http://dsi-studio.labsolver.org/).

Assessment of layer thickness of fiber tracts

30 µm thick coronal sections were made using a VT 1000 vibratome (Leica Biosystems, Nussloch, Germany) and collected in 20 % ethanol. Sections were mounted on SuperFrost slides (R. Langenbrinck, Emmendingen, Germany) and air-dried overnight. The next day, sections were rinsed three times in phosphate buffered saline (PBS).

In case of sections of the forebrain, sections were incubated in a solution containing antibodies directed against CNPase (No. PA5-19551, Thermo Fisher Scientific, Waltham, MA, USA) 1:300 in 3 % normal goat serum (NGS) and 0.1 % Triton-X100 (Serva, Heidelberg, Germany) in PBS) at 40 °C for 24 h. After rinsing in PBS (three times for 5 min at room temperature (RT)), sections were incubated in a solution containing Alexa Flour 488 conjugated goat anti-rabbit IgG (Dianova, Hamburg, Germany) 1:400 in 3 % NGS and 0.1 % Triton-X100 in PBS) for 2 h at RT. Sections were rinsed in PBS (three times for 5 min at RT) and afterward incubated in a solution containing Molecular Probes 4’,6-diamidino-2-phenylindole (DAPI, Thermo Fisher Scientific) dissolved 1:10,000 in PBS in the presence of 0.1% Triton-X100. Thereafter, sections were rinsed three times and mounted in Mowiol fluorescent mounting medium (Merck, Darmstadt, Germany).

In case of sections from the cerebellum, sections were incubated in a solution containing antibodies directed against Purkinje-cell protein 4 (Pcp4, No. sc-74816, Santa Cruz Biotechnology, Dallas, TX, USA) as described previously in detail[5]. In brief, sections were incubated in a solution (0.1 Triton X-100 and 3% serum in PBS) containing Pcp4 antibodies over night at 4 °C. Visualization was done using Cy3-conjugated secondary antibodies (Jackson Immunoresearch, West Grove, PA, USA). Sections were counterstained with DAPI (1:10,000), washed and then coverslipped in Mowiol fluorescent mounting medium.

The thickness of different brain structures was measured on a series of serial sections. Sampling was started at Bregma -1.94 mm and six consecutive sections were sampled using an Olympus BX 63 (Olympus, Tokyo, Japan) and a DP80 camera (Olympus), connected to a personal computer. Measurements were done using the software cellSense 1.13 (Olympus). Per section the thickness of the different structures (primary somatosensory cortex, hippocampal area CA1 (stratum oriens, stratum pyramidale, stratum radiatum and lacunosum-moleculare), dentate gyrus (DG, stratum moleculare and stratum granulare) molecular-, Purkinje cell - and molecular layer of the cerebral cortex as well as the thickness of the corpus callosum, alveus and external capsule. For analysis, six consecutive sections were analyzed. In each section both hemispheres were analyzed and each structure was measured on three positions – thus individual 36 measurements per region and animal were made.

Analysis of adult hippocampal neurogenesis

To analyze cell proliferation within the dentate gyrus, phosphohistone H3 was used as a specific marker; doublecortin was used to label newly formed neurons (for details see e.g. Poser et al.[6]). For determination of apoptotic cells, we used antibodies directed against cleaved (activated) caspase 3[7]. Coronal sections of 30 µm thickness were cut using a vibratome. The following antibodies and substances were used: rabbit α-phosphohistone H3 (No. sc-8656-R, Santa Cruz Biotechnology), goat α-doublecortin (1:200; sc-8066, Santa Cruz Biotechnology), rabbit α-cleaved caspase 3 (1:250, AB3623, Millipore, Merck), biotinylated horse α-goat, (1:200; Vector Laboratories, Burlingame, CA, USA), biotinylated goat α-rabbit (1:200; Vector Laboratories); Cy3-conjugated streptavidin (1:1,000; Jackson Immunoresearch).

In case of phosphohistone H3 staining and cleaved caspase 3 staining, sections were mounted and air-dried over night. On the next day, sections were washed and then incubated in sodium citrate buffer (pH 6.0) for 20 min using a microwave oven (700 W) for antigen retrieval. After this, sections were washed in a solution containing 0.1 M PBS, 0.3 % Triton X-100, 3 % bovine serum albumin (BSA) for 1 h at RT. For doublecortin immunohistochemistry, free-floating sections were used. Sections were incubated for 1 h in a blocking solution containing 0.3% Triton X-100 and 3% BSA in PBS. Thereafter, sections were incubated in a solution (0.1 Triton X-100 and 3% BSA in PBS) containing antibodies directed either against phosphohistone H3, cleaved caspase 3 or doublecortin over night at 4 °C. Visualization was done using biotinylated secondary

antibodies and Cy3-conjugated streptavidin. Sections were counterstained with DAPI (1:10,000), washed and coverslipped in Mowiol fluorescent mounting medium.

To estimate the number of the labeled cells, cell counts were made using the serial sections. Countings were performed according to the Abercrombie's correction formula (starting at ~ Bregma −1.06 mm), since this method renders biases within the range of the optical disector by taking into account that the particles counted are small compared with the section thicknes[8]. No guard zones were used, since the use of guard zones can bias even optical disector counting[9]. The Linderstrom-Lang/Abercrombie (LLA) equation for estimating numerical neuronal densities is:

$$N = n * (t/(t + H)) \text{ or } N/n = f = t/(t + H)$$

N is an estimate of the number of objects in the defined region, n is the counted number of objects, t is the mean thickness of the virtual section, H is the mean height of the objects, and f is the conversion factor for converting n to N.

In a first step, n was quantified using an Olympus BX 63 microscope fitted for fluorescence. In a second step, H, the height of the cells in the z-axis, was estimated using a computer-driven motorized stage (Märzhäuser Wetzlar, Wetzlar, Germany) connected to the Axioplan 2 imaging microscope (Carl Zeiss Microscopy, Jena, Germany) under the control of Neurolucida software (MBF Bioscience, Williston, VT, USA).

Determination of Purkinje cell density

In 30 µm thick sections of the cerebellum Purkinje cells were visualized with Pcp4 (see above). A region-of-interest (ROI, dimension: 600 x 600 µm) was superimposed on 6th cerebellar lobule (starting at ~ Bregma -6.6 mm) and the number of Purkinje cell profiles was determined. Only profiles were counted that have a clear shape of a Pcp4 stained soma.

Immunocytochemistry and image analysis

Embryonic day 16.5 WT mouse hippocampi were dissected in ice-cold HBSS medium (No. 14170–088, Gibco, Thermo Fisher Scientific), digested with 0.25% trypsin (Invitrogen, Thermo Fisher Scientific) in HBSS medium for 13 min at 37 °C, dissociated by trituration and plated on glass coverslips coated with 0.2 mg / ml poly-L-lysine hydrobromide (Sigma-Aldrich) in 0.1 M borate buffer (boric acid / sodium tetraborate). Neurons were seeded at a density of ~550 cells / mm^2 in MEM medium (No. M4655, Sigma-Aldrich) containing 10% horse serum (Thermo Fisher Scientific, 26050070) and 0.6 % glucose (No. G8769, Sigma-Aldrich) and cultured at 37 °C in a humidified incubator (5% CO_2). After 2h, medium was replaced with Neurobasal medium (No. 21103-049, Gibco, Thermo Fisher Scientific) supplemented with B27 (No. 17504-044, Gibco, Thermo Fisher Scientific) and 1 mM L-glutamine (No. 25030-081, Gibco, Thermo Fisher Scientific). Cultured hippocampal neurons were transfected at 7 days in vitro using Lipofectamine 3000 (Thermo Fisher Scientific).

Cultured hippocampal neurons were fixed 6 h after transfection by immersion in phosphate buffered saline (PBS) containing 4% paraformaldehyde for 10 min at RT. After rinsing in PBS, cells were permeabilized with 0.2% Triton X-100 in PBS containing 10% NDS for 5 min at RT. Cell cultures were incubated with the following primary antibodies diluted in PBS containing 10% NDS for 2h at RT: Chicken anti-MAP2 (No. ab5392, Abcam, Cambridge, United Kingdom), mouse anti-Ankyrin G (No. 75-147, Antibodies Incorporated, Davis, CA, USA). After washing with PBS, cells were incubated with the following secondary antibodies for 45 min at RT: Donkey anti-chicken IgY Dylight 405 (No. 703-475-155, Jackson Immunoresearch Laboratories, West Grove, PA, USA), donkey anti-mouse IgG Alexa Fluor 647 (No. A31571, Thermo Fisher Scientific). Coverslips were mounted with Dako Fluorescence Mounting Medium (No. GM30411-2, Agilent, Santa Clara, CA, USA). Images were captured by confocal laser scanning microscopy (LSM 700, Carl Zeiss Microscopy, Jena, Germany) using a 40x objective with a numerical aperture of 1.3. For axon / dendrite ratio quantification, images were analyzed with ImageJ 1.48i[10] as described previously[11]. mCherry and GFP fluorescence intensity was measured in dendrite or axon segments at a distance of 100 µm from the cell soma. After background subtraction in both channels, mCherry intensity was normalized to GFP intensity and axon/dendrite ratios calculated.

Plasmids

Human PIANP (No. RC207868, Origene Technologies, Rockville, MD, USA) was C-terminally tagged with mCherry and subcloned into the pCI vector containing the human synapsin promoter. For GFP expression, plasmid pEGFP-N1 (Clontech Laboratories, Mountain View, CA, USA) was used.

Cloning and transfection of MEF cells

Cloning, transduction and preparation of MEF cells were preformed as described previously[12].

BrdU incorporation proliferation assay

To investigate the proliferation BrdU incorporation in DNA synthesizing cells was measured. Cells were seeded on 6-well plate (50000 cells/ well). Next day, the cells were serum starved for 16 h. BrdU (10 µM) was added to the cells in conditioned media and incubated for 1 h at 37°C. After the incubation the cells were harvested, transferred into FACS tube and centrifuged for 5 min at 1200 rpm. The supernatant was discarded. 10 µl of 70% Ethanol (pre-cooled at -20°C) was added drop wise to the cells while vortexing. The cells were incubated for 20 min at RT, washed with 1 ml washing buffer and centrifuged for 5 min at 1200 rpm. The supernatant was discarded, the cells were resuspended in 300 µl 2M HCl and incubated for 20 min at RT. The cells were washed with washing buffer and centrifuged for 5 min at 1200 rpm. The supernatant was discarded, the cells were resuspended in 1 ml 0.1 M sodium borate buffer ($Na_2B_4O_7$, 10 H_2O). The cells were incubated for 2 min at RT, washed with 1 ml washing buffer and centrifuged 5 min at 1200 rpm. BrdU FITC and mouse IgG FITC were diluted 1:20 with dilution buffer. 100 µl of the diluted antibodies were added into the cells and mixed by

vortexing. The cells were incubated for 25 min in the dark at RT, washed with 1 ml washing buffer and centrifuged for 5 min at 1200 rpm. The supernatant was discarded, cells were resuspended in 1 ml PBS and analyzed with BD FACSCanto II with BD FACSDiva 6.0 software.

xCELLigence DP-based adhesion assay

E-16 plates (Roche Diagnostics, Rotkreuz, Switzerland) were coated overnight at 4°C with Fibronectin at 5 µg/ml concentration. The wells were blocked by incubating for 30 min at RT with 1 % BSA. Transduced MEF cells which were grown in T75 flask of at least ~ 80 % confluency were detached with Trypsin/EDTA. However, the cells were allowed to regenerate cell surface proteins as the detached cells were incubated at 37°C for 2-3 h in a roller. E-16 plates were equilibrated at 37°C using the xCELLigence DP impedance-based cell analysis system (Roche Diagnostics). 10000 cells/ well of MEF cells were added. The plate was allowed to stay at RT for 30 min to let the suspended cells settle down. Immediately following that the experiment was started. As the cells start to adhere to the bottom surface of the wells the electrical impedance between the microelectrode changes resulting in a change of the Cell Index. Rate of change (slope in Cell Index/h) was calculated using the xCELLigence software (Roche Diagnostics).

Plate reader-based migration assay

96 well plate was coated overnight at 4°C with the Fibronectin at 5 µg/ml concentration. The wells were blocked by incubating for 30 min at RT with 1% BSA. Transduced MEF cells which were grown in T75 flask of at least ~80% confluency were detached with Trypsin/EDTA. However, the cells were allowed to regenerate cell surface proteins as the detached cells were incubated at 37°C for 2-3 in a roller. 50000 cells/ well of MEF cells were added. The plate was allowed to stay at RT for 30 min to let the suspended cells settle down. Immediately after that the plate was incubated at 37°C for 1 h. Non-adhered cells were removed by washing the plate thrice. 50 µl of crystal violet solution (0.5% crystal violet in 20% MeOH) was added and incubated for 15 min at room temperature in darkness. The plate was washed and air-dried overnight in darkness. Crystal violet was extracted by adding 100% MeOH and shaking the plate for 15 min at RT in darkness. Equal volume of crystal violet solution was transferred into a new clean 96 well plate. Absorbance values were measured at 570 nm using Tecan Infinite 200.

xCELLigence DP-based transwell migration assay

CIM-16 plates (Roche Diagnostics) were coated 1-2 h at RT with Fibronectin at 5 µg/ml concentration. The transwells were blocked by incubating for 30 min at RT with 1% BSA. Transduced MEF cells which were grown in T75 flask of at least ~80% confluency were detached with Trypsin/EDTA. However, the cells were allowed to regenerate cell surface proteins as the detached cells were incubated at 37°C for 2-3 h in a roller. CIM-16 plates were equilibrated at 37°C using the xCELLigence DP impedance-based cell analysis system (Roche Diagnostics). Serum containing medium were added to the lower wells of the transwells. The CIM-16 plate was assembled, i.e. the upper wells were inserted into the lower wells and were fixed together. 50000 cells/ well of MEF cells in serum free

media were added in the upper wells. The plate was allowed to stay at RT for 30 min to let the suspended cells settle down. Immediately following that the experiment was started. As the cells start to migrate from the upper surface to the bottom surface of the transwells the electrical impedance between the microelectrode changes resulting in a change of the Cell Index. Rate of change (slope in Cell Index/h) was calculated using the xCELLigence software (Roche Diagnostics).

Crystal violet-based transwell migration assay

BD transwell inserts for 6 well plate were coated for 1-2 h at RT with Fibronectin at 5 µg/ml concentration. The transwell inserts were blocked by incubating for 30 min at RT with 1 % BSA. Transduced MEF cells which were grown in T75 flask of at least ~80% confluency were detached with Trypsin/EDTA. However, the cells were allowed to regenerate cell surface proteins as the detached cells were incubated at 37°C for 2-3 h in a roller. After this the BD transwell inserts were placed in a special BD 6 well plate with low evaporation lid and serum containing medium were added per well of the 6 well plate. 50000 cells/ well of MEF cells in serum free media were added in the transwell inserts. The plate was allowed to stay at RT for 30 min to let the suspended cells settle down. Following this the cells were incubated at 37 °C for 6 h. Adherent cells in the upper surface of the transwell inserts were removed by using cotton swab. Cells migrated to the bottom surface of the transwell inserts were fixed using 4% PFA at RT for 10 min. The bottom surface was stained with crystal violet solution (0.5% crystal violet in 20% MeOH) for 15 min at RT in darkness. Crystal violet solution was removed and the surface was washed several times with distilled water and allowed to air-dry overnight or longer at RT in darkness. Pictures of the crystal violet stained cells on the bottom surface of the transwell inserts were taken using camera attached to the inverse microscope and quantified further.

Primers

The following primers were used for genotyping of PianpKO mice: forward primer: ACCTTGAGGCCCTTCCTGTTTGGA; reverse primer: CTGCGCCGCTTCTGGCTACGGT (Metabion, Planegg, Germany). The following primers were used for qRT-PCR: Avp-F CTCCGCTTGTTTCCTGAGCCT, Avp-R GGTCCGAAGCAGCGTCCT, Erdr1-F GGTCAAGATGTATGTGCCACC, Erdr1-R GCTTCTACGTGTGTGCTTTCG, Pianp-F CCCTTCCTGTTTGGAGGGCGT, Pianp-R CCGCTTCTGGCTACGGTCCCA, Scn4b-F AAAGGCCACCACCATCTACG, Scn4b-R TCCCGTCGATGAGAATCCTG, b-Actin-F ACCCGCGAGCACAGCTTCTTTG, b-Actin-R, CTTTGCACATGCCGGAGCCGTTG (Metabion).

Neurochemistry

Dissected brain regions were homogenized in an extraction solution (0.1 M perchloric acid, 1 mM EDTA) using a tissue homogenizer Mixer Mill (Qiagen, Hilden, Germany) and yielded solutions subsequently centrifuged at 15 000 g for 10 min at 4 °C. In all, 10 ml of the spun sample was loaded on a high-performance liquid chromatography (HPLC) system with electrochemical detection as described previously[13]. Brain region

specific accumulation of monoamine neurotransmitters was determined by normalizing the quantified neurotransmitter amounts to the respective weight of the tissue sample.

Open field test (OFT)

OFT was conducted as described previously[13]. Locomotor activity was quantified using the TruScan system (Coulbourn Instruments, Whitehall, PA, USA). Mice were individually placed into the center area of an open field chamber (26 x 26 x 38 cm, length x width x height), equipped with 2 banks of photobeam sensors to record both the horizontal and the vertical movement of the mouse. Each mouse was recorded for 30 min under red light illumination.

Elevated plus maze test (EPM)

EPM was conducted as described previously[13]. Mice were individually placed on the center square of the plus maze facing an enclosed arm, and allowed to freely explore the maze for 5 min. Their behavior was recorded and analyzed by the video tracking software EthoVision 3.0 (Noldus, Wageningen, Netherlands).

Tail suspension test (TST)

TST was conducted as described previously[13]. Within the 5 min of testing, all movements of the mice were automatically recorded by the video tracking software EthoVision 3.0.

Forced swim test (FST)

FST was conducted as described previously[13]. Within the testing period of 5 min, the activity of each mouse was recorded from the side by the video tracking software EthoVision 3.0. Immobility was defined as motionless floating in water, only allowing movements necessary for the animal to keep its head above the water.

Object exploration test (OET)

OET was conduted according to Blick et al[14]. Mice were habituated to the room and to the open field chamber for 30 min 1 day before the experiment. On the testing day, each mouse was placed in the testing arena, a clear plastic rectangular chamber (20 ×22 x 42 cm), without objects for 5 min immediately prior to testing for habituation. The mouse was then returned to the home cage, and an unfamiliar object was positioned in the center of the testing arena. After an inter-trial interval of 1 min, the mouse was placed in the testing arena with the object for 5 min. Behavior was recorded with a digital video camera and analyzed by the video tracking software EthoVision 3.0, and manually by a trained and blinded observer. Presented objects made from metal or glass were tested in previous experiments to be attractive for exploration. After every trial the container and objects were washed with soap and water and dried prior to being used again.

Novel object recognition (ORT) and Object relocation tests (OLT)

ORT and OLT were performed according to Bevins and Besheer[15]. Testing conditions and behavior evaluation were conducted as in the OET. Each mouse was placed in the testing arena containing two objects for 5 min for habituation (sampling period). The mouse was then returned to the home cage. For ORT, one object in the testing arena was replaced with a novel object. For OLT repositioned within the testing arena. After an intertrial interval of 30 min the mouse was placed in the testing arena for 5 min (testing period). The order and the side of the object replacement or repositioning were randomized.

Nest building test (NBT)

NBT was performed according to Deacon[16]. Mice were given a cotton nestlet (5 x 5 cm) in their home cage. As a natural behavior, mice form nests out of these cotton nestlets. The nature of the nest was graded from 1 to 5, 1 being only few scratches in the nestlet and 5 being a complete nest. The observation times were 5 and 24 h after giving the nestlet.

Odor discrimination test (ODT)

ODT was performed as described by Zou et al.[17] in two phases: nonsocial odor and social odor discrimination. A piece of cotton swab was glued to a weighing cup. Odors were presented to each mouse via fresh cotton swabs (10 µl/sample) for 2 min. During the test, mice were allowed to smell oil, vanilla, orange (diluted 1:20 in oil), water, male mice urine and female mice urine (diluted 1:20 in water).

Social Interaction Test

The social interaction test apparatus was a rectangular, three-chambered box where each chamber was 20 × 22 × 42 cm. Dividing walls were made from clear plastics with small rectangular openings connecting the chambers (7 cm × 10 cm). The test mouse was first placed in the middle chamber and allowed to explore for 5 min. The rectangular openings into the two side chambers were obstructed by plastic doors during this habituation phase. After the habituation period an unfamiliar C57BL/6N male (social partner 1), which had no prior contact with the subject mouse, was placed in one of the side chambers into a small, round wire cage (approximately 11 cm in height, with a bottom diameter of 10.5 cm), which allowed nose contact between the bars, but prevented any further interaction. An empty wired cage was placed into the opposite side chamber. The location of social partner 1 in the left vs. right side chamber was randomized between trials to avoid any side specific bias. For testing, both doors to the side chambers were then opened simultaneously and the test mouse was allowed to explore the entire chamber starting from the middle chamber for a 10 min session which is denoted as sociability test. At the end of the first 10 min, each test mouse was placed back into its home cage. Another second unfamiliar C57BL/6N male (social partner 2), which had no prior contact with the subject mouse, was placed into the empty wired cage side. Then the subject mouse was put back into the middle chamber for another 10 min session which is denoted as preference for social novelty test. The test sessions were recorded with the video tracking software EthoVision 3.0 (Noldus, Wageningen, Netherlands).

Grip Strength Assessment

Mice were allowed to grab a t-bar attached to a force meter with their forepaws. The mice were gently pulled away until they released the t-bar. The grip strength was measured in gram. The mean of 6 trials was used for analysis. Grip strength was assessed on 2 following days.

Rotarod Performance Test

Mice were placed on an immobile rotarod cylinder (TSE Systems, Bad Homburg, Germany) which was then accelerated for 3 min, from 2.5 to 25 rpm. The time until the mice fell off the rotating cylinder was recorded. The mean of 3 trials was used for analysis. The test was performed on 4 following days.

Light-Dark Box Test

Light-dark box test was conducted as described previously[13]. The test apparatus consisted of two plastic chambers, connected by a tunnel of 5 x 7 x 10 cm. The dark chamber made of black plastic (20 x 15 x 30 cm) and was covered by a lid. The lit compartment made of white plastic (30 x 15 x 30 cm). Before testing, mice were habituated with the experiment room for 30 min, illuminated from above with tubular fluorescent lamps (20 lux). During testing, the lit compartment was brightly illuminated from above with tubular fluorescent lamps (600–800 lux). At the beginning of the test, mice were individually placed into the dark compartment and their behavior was monitored by the video tracking software EthoVision 3.0 (Noldus, Wageningen, Netherlands) for 5 min.

Sucrose Preference Test

Sucrose preference test was conducted as described previously[13]. Mice were initially trained for 5 consecutive days to have access to two low seeping bottles for 1 h twice a day. During initial training, one bottle was filled with 7 % (w/v) sucrose solution; the other bottle contained tap water. To prevent any side preference of the mice the location of each choice bottle was randomized and the regular water bottle was positioned to the middle of the cage lid. After 5 successive days of training, the mice preference to sucrose solutions of different concentrations (0.5 %, 1 % or 2 %) was assessed for 3 consecutive days in a total of 3 conditions. Each condition was presented twice to the animals on the particular day in the identical way as during training. The consumption of each sucrose solutions was determined by the weight change of the respective bottles. Preference ratio was calculated as (sucrose solution consumed) / (sucrose solution consumed + tap water consumed).

Novelty Induced Hypophagia Test

Novelty induced hypophagia test was conducted as described previously[13]. Mice were first trained to consume sweetened condensed milk (1:3 dilution of Milchmädchen (Nestle, Vevey, Switzerland)) in their home cage once a day for 1 h. The latency to start consuming milk and the consumption of milk within the first 10 min and the entire

training session (60 min) were assessed for each individual mouse. Training was continued until the latency to start consuming milk was below 20 s. Following the training, the sweetened condensed milk was presented to each individual mouse within a novel environment (a new cage with a metal floor) for 10 min for a second assessment.

Fear Conditioning

Fear conditioning was conducted as described previously[18]. During the training day mice were placed in the testing chamber (25 x 25 x 25 cm, Coulbourn Instruments, Whitehall, PA, USA) and were allowed to freely explore it. The testing chamber was placed inside a soundproof chamber (67 x 53 x 55 cm, Coulbourn Instruments). 2 min after the start of the conditioning session, an auditory cue (80 dB) was presented for 2 min, that terminated by a scrambled foot-shock of 0.7 mA intensity for 2 s. This sequence was repeated in total 5 times before mice were removed from the testing chamber. Freezing behavior was recorded using Freezeframe software (Coulbourn Instruments) by which freezing was defined as lack of movement except that required for respiration. 24 h after conditioning, a contextual fear memory was assessed in the testing chamber for 5 min in the absence of any auditory cue. Approximately 3 h later cue-dependent fear memory (for the same set of mice) was quantified by placing the mouse into a novel chamber (59 x 19 x 24 cm, Coulbourn Instruments) for 4 min in total. After 2 min the auditory cue (80 dB) associated with the shock was presented for 2 min. Freezing was scored using time sampling (60 s interval).

Startle and PPI

Mice were placed into a SR-LAB startle response system (San Diego Instruments, Bilaney Consultants, Duesseldorf, Germany). After a 5 min habituation phase 45 trials were presented randomly a 40 ms acoustic pre-pulse of several pre-pulse levels (72, 76, 80, and 84 dB) followed by a 40 ms 115 dB acoustic startle pulse. At the beginning the startle pulse was presented 5 times for habituation. Control measurements were performed after the startle pulse without prepulse, the prepulse without a startle pulse and without any pulse. The mean interval between the different pulses was 30 sec.

Amphetamine Challenge

Mice were habituated to the experimental room for 30 min. Then the mice were placed in TruScan system open field chamber (27 cm x 27 cm x 39 cm, Coulbourn Instruments) individually and basal locomotor activities (moving episodes and time, total distance moved, speed and jumps) were measured for 30 min. Then, subject mice were intraperitoneally injected with 100 µl of 0.9 % NaCl solution and subsequently placed in open field chamber. Locomotor activities were measured for next 30 min to have the mock injection data (control). D-amphetamine hemisulfate (Merck) was dissolved in 0.9 % NaCl solution and intraperitoneally injected into the subject mice at the dose of 2.5 mg/kg. Locomotor activities were measured for next 120 min. Locomotor activities were measured using time sampling (5 min interval) by TruScan software (Coulbourn Instruments).

Supplementary References

1 Bracke A, Schäfer S, von Bohlen und Halbach V, Klempin F, Bente K, Bracke K *et al.* ATP6AP2 over-expression causes morphological alterations in the hippocampus and in hippocampus-related behaviour. *Brain Struct Funct* 2018; **223**: 2287–2302.

2 Sidman RL, Kosaras B, Misra B, Senft S. High Resolution Mouse Brain Atlas. http://www.hms.harvard.edu/research/brain/ (accessed 16 Jan2018).

3 Bai J, Trinh TLH, Chuang K-H, Qiu A. Atlas-based automatic mouse brain image segmentation revisited: model complexity vs. image registration. *Magn Reson Imaging* 2012; **30**: 789–798.

4 Bakker R, Tiesinga P, Kötter R. The Scalable Brain Atlas: Instant Web-Based Access to Public Brain Atlases and Related Content. *Neuroinformatics* 2015; **13**: 353–366.

5 Renelt M, von Bohlen und Halbach V, von Bohlen und Halbach O. Distribution of PCP4 protein in the forebrain of adult mice. *Acta Histochem* 2014; **116**: 1056–1061.

6 Poser R, Dokter M, von Bohlen und Halbach V, Berger SM, Busch R, Baldus M *et al.* Impact of a deletion of the full-length and short isoform of p75NTR on cholinergic innervation and the population of postmitotic doublecortin positive cells in the dentate gyrus. *Front Neuroanat* 2015; **9**: 63.

7 Freund M, Walther T, von Bohlen und Halbach O. Effects of the angiotensin-(1-7) receptor Mas on cell proliferation and on the population of doublecortin positive cells within the dentate gyrus and the piriform cortex. *Eur Neuropsychopharmacol* 2014; **24**: 302–308.

8 von Bartheld C. Counting particles in tissue sections: choices of methods and importance of calibration to minimize biases. *Histol Histopathol* 2002; **17**: 639–648.

9 Baryshnikova LM, von Bohlen und Halbach O, Kaplan S, von Bartheld CS. Two distinct events, section compression and loss of particles ('lost caps'), contribute to z-axis distortion and bias in optical disector counting. *Microsc Res Tech* 2006; **69**: 738–756.

10 Schneider CA, Rasband WS, Eliceiri KW. NIH Image to ImageJ: 25 years of image analysis. *Nat Methods* 2012; **9**: 671–675.

11 Biermann B, Ivankova-Susankova K, Bradaia A, Abdel Aziz S, Besseyrias V, Kapfhammer JP *et al.* The Sushi domains of GABAB receptors function as axonal targeting signals. *J Neurosci* 2010; **30**: 1385–1394.

12 Biswas S, Adrian M, Weber J, Evdokimov K, Winkler M, Géraud C. Posttranslational proteolytic processing of Leda-1/Pianp involves cleavage by MMPs, ADAM10/17 and gamma-secretase. *Biochem Biophys Res Commun* 2016; **477**: 661–666.

13 Berger SM, Weber T, Perreau-Lenz S, Vogt MA, Gartside SE, Maser-Gluth C *et al.* A functional Tph2 C1473G polymorphism causes an anxiety phenotype via compensatory changes in the serotonergic system. *Neuropsychopharmacology* 2012; **37**: 1986–1998.

14 Blick MG, Puchalski BH, Bolanos VJ, Wolfe KM, Green MC, Ryan BC. Novel object exploration in the C58/J mouse model of autistic-like behavior. *Behav Brain Res* 2015; **282**: 54–60.

15 Bevins RA, Besheer J. Object recognition in rats and mice: a one-trial non-matching-to-sample learning task to study 'recognition memory'. *Nat Protoc* 2006; **1**: 1306–1311.

16 Deacon RMJ. Assessing nest building in mice. *Nat Protoc* 2006; **1**: 1117–1119.

17 Zou J, Wang W, Pan Y-W, Lu S, Xia Z. Methods to measure olfactory behavior in mice. *Curr Protoc Toxicol* 2015; **63**: 11.18.1-21.
18 Strekalova T, Zörner B, Zacher C, Sadovska G, Herdegen T, Gass P. Memory retrieval after contextual fear conditioning induces c-Fos and JunB expression in CA1 hippocampus. *Genes Brain Behav* 2003; **2**: 3–10.
